# Reduction of the RNA Binding Protein TIA1 Exacerbates Neuroinflammation in Tauopathy

**DOI:** 10.3389/fnins.2020.00285

**Published:** 2020-04-07

**Authors:** Chelsey Jenna LeBlang, Maria Medalla, Nicholas William Nicoletti, Emma Catherine Hays, James Zhao, Jenifer Shattuck, Anna Lourdes Cruz, Benjamin Wolozin, Jennifer Irene Luebke

**Affiliations:** ^1^Laboratory of Cellular Neuroscience, Department of Anatomy & Neurobiology, Boston University School of Medicine, Boston, MA, United States; ^2^Laboratory of Neurodegeneration, Department of Pharmacology & Experimental Therapeutics, Boston University School of Medicine, Boston, MA, United States; ^3^Department of Neurology, Boston University School of Medicine, Boston, MA, United States; ^4^Department of Neuroscience, Boston University, Boston, MA, United States

**Keywords:** tauopathy, neuroinflammation, microglia, hippocampus, RNA binding protein, neurodegeneration

## Abstract

Neuroinflammatory processes play an integral role in the exacerbation and progression of pathology in tauopathies, a class of neurodegenerative disease characterized by aggregation of hyperphosphorylated tau protein. The RNA binding protein (RBP) T-cell Intracellular Antigen 1 (TIA1) is an important regulator of the innate immune response in the periphery, dampening cytotoxic inflammation and apoptosis during cellular stress, however, its role in neuroinflammation is unknown. We have recently shown that TIA1 regulates tau pathophysiology and toxicity in part through the binding of phospho-tau oligomers into pathological stress granules, and that haploinsufficiency of TIA1 in the P301S mouse model of tauopathy results in reduced accumulation of toxic tau oligomers, pathologic stress granules, and the development of downstream pathological features of tauopathy. The putative role of TIA1 as a regulator of the peripheral immune response led us to investigate the effects of TIA1 on neuroinflammation in the context of tauopathy, a chronic stressor in the neural environment. Here, we evaluated indicators of neuroinflammation including; reactive microgliosis and phagocytosis, pro-inflammatory cytokine release, and oxidative stress in hippocampal neurons and glia of wildtype and P301S transgenic mice expressing TIA1^+/+^, TIA1^+/–^, and TIA1^–/–^ in both early (5 month) and advanced (9 month) disease states through biochemical, ultrastructural, and histological analyses. Our data show that both TIA1 haploinsufficiency and TIA1 knockout exacerbate neuroinflammatory processes in advanced stages of tauopathy, suggesting that TIA1 dampens the immune response in the central nervous system during chronic stress.

## Introduction

Neuroinflammation exacerbates pathology in neurodegenerative diseases, including tauopathies such as Alzheimer’s Disease (AD) and Frontotemporal dementia (reviews: Mrak and Griffin, 2005; Block et al., 2007; Lull and Block, 2010; Perry et al., 2010; Cunningham, 2013; Laurent et al., 2018; Ransohoff and El Khoury, 2019). In tauopathies, the microtubule associated protein tau becomes hyperphosphorylated (as phospho-tau) and aggregates within neurons and glia. While tau is normally predominantly present in neuronal axons, phospho-tau mislocalizes to the somatodendritic compartment, where it aggregates into toxic oligomers, fibrils, and eventually neurofibrillary tangles (NFTs) (reviews: Lee et al., 2001; Iqbal et al., 2005; Ballatore et al., 2007; Kahlson and Colodner, 2015; Wang and Mandelkow, 2016). The presence of phospho-tau in the neuropil primes microglia -the resident antigen-presenting cells of the central nervous system- causing them to shift from a homeostatic to a reactive state (reviews: Maccioni et al., 2010; Zilka et al., 2012; Cunningham, 2013; Barron et al., 2017; Leyns and Holtzman, 2017; Laurent et al., 2018).

Reactive microglia perform phagocytosis and antigen-presentation, release anti-inflammatory and/or pro-inflammatory mediators, and produce reactive oxygen species (ROS) making them both potentially protective and potentially harmful during disease progression (reviews: Lynch, 2009; Ransohoff and Perry, 2009; Glass et al., 2010; Lull and Block, 2010; Perry et al., 2010; Cunningham, 2013; Li and Barres, 2018). A positive relationship between levels of microglial reactivity and tau-mediated pathogenesis has been characterized in the brains of both human patients and mouse models of tauopathy (Serrano-Pozo et al., 2011, reviews: Barron et al., 2017; Leyns and Holtzman, 2017; Laurent et al., 2018; Perea et al., 2018). Reactive microgliosis begins in early stages of disease, with microglial infiltration and proliferation in neural regions where phospho-tau first accumulates, such as the entorhinal cortex and hippocampus (Bellucci et al., 2004, 2011; Yoshiyama et al., 2007; Sasaki et al., 2008; Maphis et al., 2015). Although initially neuroprotective, the sustained release of pro-inflammatory mediators by reactive microglia exacerbates neurodegeneration in tauopathy, which in turn increases the neuroinflammatory response, in a progressively detrimental feedback loop (reviews: Maccioni et al., 2010; Zilka et al., 2012; Cunningham, 2013; Barron et al., 2017; Leyns and Holtzman, 2017; Laurent et al., 2018).

We have recently demonstrated that haploinsufficiency of the RNA binding protein (RBP) T-Cell Intracellular Antigen 1 (TIA1) significantly reduces neurodegeneration in the P301S mouse model of tauopathy, however the underlying mechanism(s) of action are not known (Apicco et al., 2017; Maziuk et al., 2018; Jiang et al., 2019). Given the close interplay between neuroinflammatory and neurodegenerative processes we reasoned that one mechanism by which TIA1 reduction ameliorates tau pathology could be through modulation of the inflammatory response. In support of this idea, TIA1-mediated stress granule formation in microglia significantly reduces their phagocytic capabilities; thus, reduction of TIA1 would be expected to enhance protective microglial phagocytic activity in tauopathy (Ghosh and Geahlen, 2015). There is also evidence to suggest that TIA1 reduction increases detrimental inflammatory responses. TIA1 is found in virtually all cell types, where it participates in both mRNA alternative splicing and transport in the nucleus (reviews: Waris et al., 2014; Sánchez-Jiménez and Izquierdo, 2015). However, during cellular stress in peripheral tissues, TIA1 is anti-inflammatory, binding and sequestering mRNA transcripts of pro-inflammatory and pro-apoptotic mediators in cytoplasmic stress granules, silencing their translation (Piecyk et al., 2000; Dixon et al., 2003; Phillips et al., 2004; Lopez de Silanes et al., 2005; Waris et al., 2014, review: Carpenter et al., 2014). Effects of TIA1 reduction on peripheral innate immunity have been well characterized, with several studies demonstrating that TIA1 knockout leads to increased production of inflammatory cytokines (i.e., TNFα, COX2, IL6) in peripheral cells and tissues (Piecyk et al., 2000; Cok et al., 2003; Dixon et al., 2003; Phillips et al., 2004; Kim et al., 2005; Karalok et al., 2014), like bone (Phillips et al., 2004) endometrium (Karalok et al., 2014), and in peritoneal macrophages (Piecyk et al., 2000). This pro-inflammatory response is heightened when challenged with an immune insult (Piecyk et al., 2000; Cok et al., 2003; Phillips et al., 2004; Kim et al., 2005; Karalok et al., 2014). Whether reduction of TIA1 has a similar effect in the central nervous system is unclear (Gong and Hewett, 2018; Rayman et al., 2019). Here, we elucidate the role of TIA1 in neuroinflammation by characterizing the effect of TIA1 haploinsufficiency and of TIA1 knockout on microgliosis, microglial phagocytosis, inflammatory cytokine production, oxidative stress, and synapse number and structure in wildtype and in early (5 month) and advanced stage (9 month) P301S mice that express TIA1^+/+^, TIA1^+/–^, and TIA1^–/–^ using molecular, histological, and ultrastructural analyses. The neuroinflammatory response was significantly higher in the hippocampus of both P301S TIA1^+/–^ and P301S TIA1^–/–^ mice compared to P301S TIA1^+/+^ mice in late-stage of disease, consistent with the idea that TIA1 dampens the neuroinflammatory response during the chronic stress of tauopathy.

## Materials AND METHODS

### Subjects

Experimental subjects included Wildtype (WT) C57BL/6J and P301S mice expressing RNA-binding protein TIA1 with full expression (+/+), haploinsufficiency (±) and knock-out (^–/–^). C57BL/6J and P301S-PS19 [B6;C3-Tg(Prnp-MAPT^∗^P301S)PS19Vle/J] mice were purchased from Jackson Laboratories. P301S mice are transgenic for the human P301S mutation of the MAPT gene found in familial Fronto-Temporal Dementia with Parkinsonism delivered via the mouse prion promoter, and backcrossed with C57BL/6j mice for haploinsufficient expression (Allen et al., 2002). TIA1^–/–^ [B6.129S2(C)-Tia1^TM 1Andp^/J] mice were generated by Anderson and colleagues and obtained from Dana Farber Cancer Institute (Phillips et al., 2004). TIA1^–/–^ mice have been extensively back-crossed with C57BL/6J animals. To generate a haploinsufficient colony, P301S mice were crossed with wildtype TIA1^–/–^ mice producing P301S^+/–^ TIA1^+/–^ and P301S^–/–^ TIA1^+/–^ pups. P301S^+/–^ TIA1^+/–^ mice were then crossed with P301S^+/–^ TIA1^+/–^ mice to generate littermates of all six genotypes (P301S^+/–^, TIA1^+/+^; P301S^+/–^, TIA1^+/–^; P301S^+/–^, TIA1^–/–^; P301S^–/–^, TIA1^+/+^; P301S^–/–^, TIA1^+/–^; P301S^–/–^, TIA1^–/–^) with the same genetic background at Mendelian genetic proportions. P301S^–/–^ mice are referred to as wildtype (WT). Mice were sacrificed at 9–9.5 months of age and 5–5.5 months of age under conditions specified below for histological and biochemical analyses. The total number of subjects used in all experimental preparations at 5 months were: WT TIA1^+/+^ *n* = 8 (2 males, 6 females), WT TIA1^+/–^ *n* = 5 (4 males 1 female), WT TIA1^–/–^ *n* = 4 (2 males, 2 females), P301S TIA1^+/+^ *n* = 8 (3 males, 5 females), P301S TIA1^+/–^ *n* = 10 (4 males, 6 females), P301S TIA1^–/–^ *n* = 9 (7 males, 2 females). The total number of subjects used in all experimental preparations at 9 months were: WT TIA1^+/+^ *n* = 8 (5 males, 3 females), WT TIA1^+/–^ *n* = 4 (3 males, 1 female), WT TIA1^–/–^ *n* = 3 (3 males), P301S TIA1^+/+^ *n* = 7 (5 males, 2 females), P301S TIA1^+/–^ *n* = 8 (8 males), P301S TIA1^–/–^ *n* = 8 (5 males, 3 females). All animals were handled according to animal care guidelines from the NIH *Guide for the Care and Use of Laboratory Animals* and *the U.S. Public Health Service Policy on Humane Care and Use of Laboratory Animals* and research procedures were approved by the Institutional Animal Care and Use Committee at Boston University School of Medicine.

#### Confirmatory Genotyping

At the time of euthanasia, tail samples were collected, tissue was digested, and DNA was extracted utilizing the Qiagen DN-easy kit. Samples were sent to Transnetyx automated genotyping, and P301S and TIA1 genotypes were confirmed.

### Perfusion and Tissue Harvesting

For immunohistochemistry and electron microscopy (EM) experiments: Subjects were deeply anesthetized via intraperitoneal injection of Socumb Solution (6 g Sodium Pentobarbital/250 mL, Henry Schein) at a 1:5 dilution in sterile saline delivered at 15 μl/2 g body weight. Next, we carried out transcardial perfusion with 50 mL of 4% paraformaldehyde (PFA) warmed to approximate body temperature (∼36°C). Calvaria were removed, and heads were post-fixed in 4% PFA overnight. Brains were then dissected from the skull-base and post-fixed in 4% PFA for 48 h (Kopeikina et al., 2011). After that, brains were dried with filter paper, weighed, and the cerebellum and rostral frontal cortex were removed. Brains were then sectioned coronally at 100 μm on a Leica VT1000S Vibratome, and stored in six series of serial sections. Sections were stored in antifreeze solution [30% ethylene glycol, 30% glycerol, 40% 0.05 M Phosphate Buffer (PB)] at −20°C.

#### For qT-PCR Biochemical Experiments

Subjects were deeply anesthetized via inhalation of Isothesia (99.9% isoflurane, Henry Schein), and transcardially perfused with 15 mL ice cold 1× PBS (Gibco). Tail samples were collected and frozen at −20°C for subsequent confirmatory genotyping, as described above. Brains were dissected from skulls, and bisected on ice. The olfactory bulb, cerebellum, and brain stem were removed, and each hemisphere was flash frozen at −80°C on dry ice. Samples were stored at −80°C until use.

### Immunohistochemistry (IHC) for Fluoresence Microscopy

#### Dual or Triple IHC Immunofluorescence Labeling

Immunohistochemical methods were adapted from protocols established in Medalla et al., 2017. Free floating sections were removed from antifreeze and rinsed with 0.01 M phosphate-buffered saline (PBS) 2 × 10 min, then incubated in 50 mM glycine for 1 h at room temperature (RT). Sections were then rinsed in 0.01 M PBS 2 × 10 min, and antigen retrieval was performed using 10 mM sodium citrate at 8.5 pH in a 65–75°C water bath for 20 min, or 10 mM sodium citrate at 6.0 pH in 85–95°C water bath for 45 min 1 h (for TNFα detection). After that, sections were rinsed in 0.01 M PBS 3 × 10 min, and were incubated for 2 h in pre-block solution [0.01 M PBS, 5% BSA, 5% Normal Donkey Serum (NDS), 0.2% Triton-x] to reduce non-specific binding of the secondary antibodies. Primary antibodies (Table 1) were diluted in incubation buffer (0.1 M PB, 0.2% BSA-c, 1% NDS, 0.1% Triton-x), and sections were incubated at 4°C for 48 h, with two microwave incubation sessions (2 × 10 min at 150 Watts) using the Pelco Biowave Pro (Ted Pella) over the 48 h period to increase antibody penetration. After rinsing (3 × 10 min in 0.01 M PBS), sections were incubated in secondary antibodies (Table 2) diluted in incubation buffer (1:200–1:250), at 4°C for 4 h, with one microwave session (2 × 10 min at 150 Watts). For optimal labeling of microglia processes, co-incubation in IBA1 and P2rY12 primary antibodies was employed, followed by incubation in biotinylated secondary anti-rabbit secondary antibodies, which was then be amplified using Streptavidin-alexa-fluor conjugates (1:200 incubation for 24 h). Sections were then rinsed 2 × 10 min in 0.01 M PBS, overnight in 0.1 M PB, and mounted with prolong anti-fade gold mounting medium (Invitrogen).

**TABLE 1:** Primary antibodies utilized in immunohistochemistry.

**Antigen**	**Abbreviation**	**Host**	**Dilution**	**Source**	**Catalog #**
8-Oxo-2′-deoxyguanosine	8-OHdG	Goat	1:500	NovusBio	NB600-1508
Interleukin-1 Beta	IL-1β	Mouse	1:500	Santa Cruz	sc-32294
Ionized Calcium Adaptor Protein 1	IBA1	Rabbit; Goat	1:250;1:1000	Wako; Abcam	019-19741; ab5076
Major Histocompatibility Complex II	MHCII	Rat	1:250	Thermofisher	14-5321-82
Phospho-Tau (Ser202, Thr205)	AT8	Mouse	1:100	Thermofisher	MN1020
Purinergic Receptor P2y12	P2ry12	Rabbit	1:250	NovusBio	NBP1-78249
Synaptophysin	SYP	Mouse	1:250	Santa Cruz	sc-17750
Tumor Necrosis Factor-alpha	TNFα	Goat	1:40	Thermofisher	PA5-46945

**TABLE 2:** Secondary antibodies utilized in immunohistochemistry.

**Antigen**	**Host**	**Conjugate**	**Source**	**Catalog #**
Goat IgG	Donkey	Alexa 488	Jackson	705-546-147
Mouse IgG	Donkey	Alexa 488	Invitrogen/Jackson	A21202
Goat IgG	Donkey	Alexa 546	Invitrogen	A11056
Mouse IgG	Donkey	Alexa 546	Invitrogen	A10034
Mouse IgG	Goat	Alexa 546	Invitrogen	A11018
Rat IgG	Donkey	Alexa 647	Jackson	712-606-150
Rabbit IgG	Donkey	Alexa 647	Invitrogen	A31573
Goat IgG	Donkey	Biotin	Jackson	705-066-147
Rabbit IgG	Donkey	Biotin	Jackson	711-066-152
Mouse IgG	Donkey	Gold (0.8 nm)	Aurion	800.322

*All diluted 1:200 or 1:250.*

#### Batch Processing

For all immunolabeling experiments visualizing IBA1/P2ry12, MHCII, AT8, and TNFα sections were processed in batches with a batch defined as one series/genotype. A series contained 3–4 100 μm sections spanning the rostral to caudal expanse of the hippocampus, with the most rostral section containing the beginning of the CA1 region. For IHC experiment visualizing IL-1β and 8-OHdG 2 100 μm sections in the mid-hippocampus (1 dorsal, 1 ventral) were processed.

#### Subjects

IHC experiment visualizing IBA1/P2ry12, MHCII, and AT8 was composed of the following subjects: 5-month cohort: WT TIA1^+/+^ *n* = 5 (1 male, 4 female), WT TIA1^+/–^ *n* = 5 (4 males 1 female), WT TIA1^–/–^ *n* = 4 (2 males, 2 females), P301S TIA1^+/+^ *n* = 5 (1 male, 4 females), P301S TIA1^+/–^ *n* = 7 (4 males, 3 females), P301S TIA1^–/–^ *n* = 6 (6 males). 9-month cohort: WT TIA1^+/+^ *n* = 5 (4 males, 1 female), WT TIA1^+/–^ *n* = 4 (3 males, 1 female), WT TIA1^–/–^ *n* = 3 (3 males), P301S TIA1^+/+^ *n* = 4 (4 males), P301S TIA1^+/–^ *n* = 5 (5 males), P301S TIA1^–/–^ *n* = 5 (5 males). IHC experiment visualizing TNFα was composed of the following the subjects: 5-month cohort: WT TIA1^+/+^ *n* = 3 (1 male, 2 females), WT TIA1^+/–^ *n* = 3 (2 males, 1 female), WT TIA1^–/–^ *n* = 3 (2 males, 1 female), P301S TIA1 +/+ *n* = 3 (3 females), P301S TIA1^+/–^ *n* = 3 (2 males, 1 female), P301S TIA1^–/–^ *n* = 3 (3 males). 9-month cohort: WT TIA1^+/+^ *n* = 3 (3 females), WT TIA1^+/–^ *n* = 3 (3 males), WT TIA1^–/–^ *n* = 3 (3 males), P301S TIA1^+/+^ *n* = 3 (3 males), P301S TIA1^+/–^ *n* = 4 (4 males), P301S TIA1^–/–^ *n* = 4 (4 males). IHC experiment visualizing IL1β, 8OHDG, IBA1 was composed of the following the subjects: 5-month cohort: WT TIA1^+/+^ *n* = 3 (1 male, 2 females), WT TIA1^+/–^ *n* = 3 (2 males, 1 female), WT TIA1^–/–^ *n* = 3 (2 males, 1 female), P301S TIA1^+/+^ *n* = 3 (1 male, 2 females), P301S TIA1^+/–^ *n* = 3 (1 male, 2 female), P301S TIA1^–/–^ *n* = 3 (3 males). 9-month cohort: WT TIA1^+/+^ *n* = 3 (3 females), WT TIA1^+/–^ *n* = 3 (2 males, 1 female), WT TIA1^–/–^ *n* = 3 (3 males), P301S TIA1^+/+^ *n* = 3 (3 males), P301S TIA1^+/–^ *n* = 3 (3 males), P301S TIA1^–/–^ *n* = 3 (3 males).

### Confocal Microscopy

Immunofluorescent (IF) labeling was detected using low-resolution and high-resolution laser scanning confocal microscopy (Leica TCS SPE confocal microscope). Low resolution images of full hippocampal anatomy were captured using a plan-fluotar 10 × 0.3 NA air objective, at 1.08 μm × 1.08 μm × 2 μm voxel size. High resolution images of the dentate gyrus were captured with a plan-apochromat 40 × 1.3 NA oil immersion objective, at 0.17921 μm × 0.17921 μm × 0.5 μm voxel size. All image stacks were captured optimized gain and offset settings, adjusted as needed with z-stack detector gain compensation to correct for uneven brightness across the z-depth of tissue, to achieve an equivalent dynamic signal range for each image stack. Images were deconvolved using AUTOQUANT3 software (Media Cybernetics), and saved as 8 bit TIFF files.

### Microglial Cell Density Estimation

#### IF and Imaging

Microglial cell membrane morphology was detected using IF, with primary antibodies against IBA1 and P2ry12 proteins, detected by the same secondary antibody fluorophore, as described above. Microglial antigen presentation was detected using IF with primary antibody against the MHCII complex. Low resolution image stacks were captured using confocal microscopy as described above, sampling the entire dorsal hippocampal formation in 2 sections/animal. High resolution image stacks were captured using confocal microscopy, as described above, with 3 fields from 3 100 μm coronal sections spanning the hippocampus captured per animal. Fields encompassed the dentate gyrus including both superior and inferior blades of the granule cell layer (GCL), the hilus, and a fraction of the superior and inferior molecular layers, just lateral to the dentate apex. After deconvolution, image stacks were imported to LSM image browser software, where the middle 30 μm of each z-stack were saved for analysis. In the rare case of an antibody penetration problem or damage, the top 30 μm of the z-stack were analyzed.

#### Cell Counting

Thirty micrometer image stacks were imported into Neurolucida software (MBF Biosciences) to quantify density of microglial cells based on morphology and degree of antigen presentation, both of which have been correlated with microglial function (Stence et al., 2001; Karperien et al., 2013; Verdonk et al., 2016; Paasila et al., 2019, reviews: Lynch, 2009; Beynon and Walker, 2012; Salter and Stevens, 2017). Microglial morphology was classified as either ramified or hypertrophic/amoeboid (H/A), with ramified cells exhibiting small round somata, and long, thin, branched processes, and hypertrophic cells exhibiting large, ballooned, ovoid somata and short thickened processes, consistent with previous literature (Stence et al., 2001; Karperien et al., 2013; Verdonk et al., 2016; Paasila et al., 2019, review: Beynon and Walker, 2012). Antigen presentation was determined based on degree of MHCII reactivity. Microglia were categorized as MHCII- if there was no MHCII reactivity, MHCII + if there was focal MHCII reactivity, and MHCII + + if there was whole-cell MHCII reactivity. Optical fractionator counting rules were applied to each image stack, as previously described (Fiala and Harris, 2001). Cell bodies touching the top and right borders of image stacks were counted, and cell bodies touching the bottom and left borders of image stacks were not counted. Cell bodies present in the first optical section of the image stack were not counted, with the top section acting as a guard zone. Cell bodies touching the last optical section of the image stack were counted, unless there was not enough information for classification.

#### Data Analysis

Neurolucida data files were exported to Neurolucida Explorer software (MBF Biosciences). Marker analysis was carried out and total microglia, total ramified microglia, total H/A MHCII- microglia, total H/A MHCII + microglia, and total H/A MHCII + + microglia were recorded. Raw data were normalized by converting total number into density (#/mm^3^); total numbers were divided by image volume in μm^3^ and subsequently multiplied by 1,000,000,000. Outlier analysis was performed on raw data sampled from all subjects within a genotypic group, and average density per subject was calculated. Outlier analysis was then performed on average densities for each subject, and averages across genotypes were compared using One-Way ANOVA with Bonferonni *Post hoc* analysis in SPSS software.

### Particle Analysis to Estimate the Expression of Phospho-Tau, Presynaptic Terminals, Inflammatory Markers, and DNA Damage

#### IF and Image Preparation

Phospho-tau (AT8), presynaptic terminal (SYP), inflammatory cytokine (TNFα, IL1-β), and DNA damage (8-OHdG) markers were detected using IF as described above, using primary antibodies in Table 1. Image stacks were captured using confocal microscopy, sampling the dentate gyrus, as described above. After deconvolution, image stacks were imported to LSM image browser software or ImageJ software, and the top 15 μm (10 μm SYP) of each z-stack were saved for analysis.

#### Particle Analysis

Images were imported into ImageJ software, and scale was calibrated. Channels were split to isolate the antigen of interest, and brightness and contrast were reset. The image stack with the greatest percentage of reactive pixels for each subject was utilized to set the threshold for analysis, and that threshold ± 5 units was applied to all images sampled from the individual subject. Percent area occupied by reactive pixels was calculated. Outlier analysis was performed on raw data sampled from each image analyzed, and an average value was obtained for each field. Outlier analysis was then performed on average values for all fields sampled in a genotypic group, and averages across genotypes were compared using One-Way ANOVA with Bonferonni *post hoc* analysis in SPSS software. For AT8 experiment *n* = 12–15 fields analyzed/4–5 animals/genotype. For SYP and TNFα experiments *n* = 9–12 fields/3–4 animals/genotype. For IL-1β and 8-OHdG experiments *n* = 12 fields/3 animals/genotype. Author was blind to genotype.

### Analyses of Colocalization of Microglia and Phospho-Tau and Microglia and IL-1β

#### IF and Image Preparation

Microglial markers (IBA1/P2ry12, MHCII) and phospho-tau (AT8) or Microglia (IBA1/P2ry12) and IL-1β were detected using IF as described above, using primary antibodies in Table 1. Image stacks were captured using confocal microscopy, sampling the dentate gyrus, as described above. After deconvolution, image stacks were imported to LSM image browser software or ImageJ software, and the top 15 μm of each z-stack were saved for analysis.

#### Colocalization Analysis

We assessed colocalization of microglial markers (IBA1/P2ry12, MHCII) with phospho-tau (AT8), and microglia (IBA1/P2ry12) and IL-1β using the EZ colocalization plugin for ImageJ software (Stauffer et al., 2018). Threshold was set manually for each reporter in the middle of 15 μm image stacks, and top 10% pixel intensity was used to calculate Manders’ M1 and M2 coefficients (Manders et al., 1993; Stauffer et al., 2018). We analyzed differences in the M1 coefficient, which represented the percentage of microglial pixels (IBA1/Pry12 or MHCII) overlapped with phospho-tau pixels (AT8), or the percentage of IL-1β pixels overlapped with microglial (IBA1/P2ry12) pixels. Outlier analysis was performed on raw data sampled from each image analyzed, and an average value was obtained for each field. Outlier analysis was then performed on average values for all fields sampled in a genotypic group, and averages across genotypes were compared using One-Way ANOVA with Bonferonni *post hoc* analysis in SPSS software. For IBA1/P2ry12 and AT8 experiments *n* = 12–15 fields/4–5 animals/genotype. For IBA1/P2ry12 and IL-1β experiments *n* = 12 fields/3 animals/genotype. For visualization of colocalization in photomicrographs of Figures 2C,D, 6B,C, Colocalization Finder plugin for ImageJ was utilized on 2D maximum projections of 15μm image stacks, producing a white mask at points of colocalization.

### Correlation and Regression Analyses

Correlation and regression analyses were carried out within each P301S genotypic group at 9-months, to determine relationships between phospho-tau expression and microgliosis. Correlation and regression were performed between percent area occupied by AT8 particles and density of total microglia, as well as between percent area occupied by AT8 particles and density of MHCII++ microglia. Correlated data was collected from individual subjects, at three anatomical levels across the rostral to caudal expanse of the hippocampus (*n* = 3 fields, from each animal/genotype). Analyses were carried out utilizing the bivariate Pearson’s correlation function in SPSS software, with a significance level of *p* = 0.05. Correlation scatter plots were generated, and linear regression analysis was carried out to plot the line of best fit using the regression plots function in SPSS.

### Electron Microscopy (EM) Analyses of Synapses, Microglia-Neuron Interactions, and Mitochondrial Morphology

#### Dual Pre-embedding IHC for EM

Two tissue sections containing the dorsal hippocampus were chosen from a single series prepared from *n* = 1 per genotype (P301S^+/–^, TIA1^+/+^; P301S TIA1^+/–^ TIA1^+/–^; P301S^+/–^, TIA^–/–^, and WT TIA1^+/+^) for analysis. Free-floating sections were processed for dual pre-embedding IHC according to Medalla and Barbas (2009). After rinses in 0.01 M PBS (2 × 10 min 4°C), tissue was incubated in 50 mM glycine for 1 h at RT. After that, tissue was rinsed in 0.01 M PBS 3 × 10 min, and sections were incubated for 2 h in pre-block solution (0.01 M PBS, 5% BSA, 5% NDS, 0.025% Triton-x). Sections were incubated in primary antibodies (Table 1) diluted in incubation buffer (0.1 M PB, 0.2% BSA-c, 1% NDS, 0.025% Triton-x), overnight at 4°C. Tissue was microwaved with the Pelco Biowave Pro (Ted Pella) for 2 × 5 min at 150 Watts twice over the incubation period. Tissue was rinsed 3 × 10 min in 0.01 M PBS, and then incubated in secondary antibodies (1:200 donkey anti-goat IgG + biotin; 1:100 donkey anti-Mouse IgG + 0.8nm gold, diluted in incubation buffer containing 0.1% gelatin, Table 2) overnight at 4°C, and microwaved 1 × 10 min at 150 W during the incubation period. Tissue was then washed in 0.1 M PB with 0.2% BSA-c for 15 min, and quickly rinsed in 0.1 MPB. Then sections were incubated in 20 mM Sodium Citrate (pH 7.1) 5 min, and rinsed 1–2 min in dH2O. Sections were incubated in silver enhancement solution (Aurion) for 60–90 min at RT. Tissue was once again incubated in 20 mM Sodium Citrate (pH 7.1) for 5 min, rinsed in dH2O, and washed in 0.1 M PB (pH 7.4) 3 × 10 min. Next, tissue was incubated in avidin and biotin- horseradish peroxidase (HRP) enzyme complex solution (Vector PK6100 ABC elite kit, Vector Laboratories) first in the microwave (2 × 3 min at 150 W) and then at room temperature for 5–10 min. Tissue was then washed 3 × 10 min in 0.1 M PB, and stained using diaminobenzadine (DAB) HRP substrate (Vector Laboratories) for 4 min. To terminate this reaction sections were washed in 0.3% H2O2 in 0.1 M PB. Finally, sections are washed 3 × 10 min in 0.1 MPB, and post-fixed in a 6% glutaraldehyde, 2% paraformaldehyde solution through microwaving at 150W until sample temperature was >30°C. Sections were incubating overnight in 6% glutaraldehyde, 2% paraformaldehyde solution at 4C, as previously described (Medalla and Barbas, 2009).

#### EM Processing and Embedding

Tissue was washed in 0.1 M PB 3× over 20 min, and processed and embedded for EM, as previously described (Medalla and Barbas, 2010). Sections were incubated in two-stage osmium: first in 1% Osmium with 1.5% KFe in 0.1 M PB (microwaved 2 × 2 min at 100W, 10 min at RT), followed by 1% osmium in 0.1 M PB (microwaved 2 × 2 min at 100W, 10 min at RT). Tissue was rinsed 3× in 0.1 M PB, and 2× in dH2O, followed by dehydration in increasing gradients of alcohol (3 × 5 min 50% ethanol, 30 min in 70% ethanol with 1% Uranyl Acetate, 3 × 5 min each in 90, 95, and 100%). To infiltrate resin, sections were rinsed in 100% propylene oxide (2 × 7.5 min), followed by a 1:1 propylene oxide:araldite resin (Ted Pella) solution for 3 h, and in 100% Araldite overnight in a vacuum desiccator. Tissue was then flat embedded in araldite and cured at 60°C 48 h. The apex of the dentate granule cell layer, as well as the hilus, were micro dissected and re-embedded on araldite blocks.

#### Serial Ultra Sectioning and Imaging

Resin embedded tissue blocks were cut using a Leica ultramicrotome and diamond knife, first into 1μm thick sections, which were stained with toluidine blue for confirmation of anatomical structures. A series of 20–50 ultrathin sections were cut at 50 nm and mounted on pioloform coated copper grids. Sections were photographed using JEOL 100CX transmission electron microscope at 20,000× magnification and 80kV, using Gatan digital camera software. Regions sampled consisted of 2–4 fields within the hilus, with approximate total volume equivalent. Fields were chosen based on presence of DAB positive microglial cells.

#### 3-D Structural Analysis of Microglia, Synapses, and Mitochondria

Images were imported into Adobe Photoshop software for montaging. Montaged images were then imported into Reconstruct software for analysis (Fiala, 2005). Serial images were aligned using fiducial markers (cross-sectional mitochondrial profiles), and microglial processes were traced section-by-section and reconstructed in 3-D. Appositions of microglial processes with all neuronal structures were marked and the area of contact was traced in 3D. Structures were categorized as AT8 + based on presence of gold particles. Average number of neural structure contacts/microglial surface area was calculated in each field sampled per genotype. Excitatory and inhibitory synapses were identified utilizing established criteria (Peters et al., 1991). Synapse density was quantified using 3D stereologic counting techniques, as described previously (Fiala and Harris, 2001; Medalla and Luebke, 2015). Average number of synapses were calculated for each field sampled per genotype. All mitochondria in neural and microglial structures were reconstructed in 3D, and categorized as normal or abnormal in morphology. Abnormal mitochondria were defined based on previously established profiles including; donut, curved, beads on a string, and frothy (gaps in cristae and broken outer membrane) (Yoshiyama et al., 2007; Kopeikina et al., 2011; Duboff et al., 2012; Ahmad et al., 2013; Hara et al., 2014; Long et al., 2015). Average density of abnormal microglia was calculated for volume of tissue sampled, and volume of microglia in each field per genotype. Averages across genotypes were compared using One-Way ANOVA with Bonferonni *post hoc* analysis in SPSS software, where *n* = 3–4 fields/genotype.

### qRT PCR

#### Subjects

5-month cohort: WT TIA1^+/+^ *n* = 3 (1 male, 2 females), P301S TIA1^+/+^ *n* = 3 (2 males, 1 female), P301S TIA1^+/–^ *n* = 3 (3 females), P301S TIA1^–/–^ *n* = 3 (1 male, 2 females). 9-month cohort: WT TIA1^+/+^ *n* = 3 (1 male, 2 females), P301S TIA1^+/+^ *n* = 3 (1 male, 2 females), P301S TIA1^+/–^ *n* = 3 (3 males), P301S TIA1^–/–^ *n* = 3 (3 females). Right and Left hemispheres were utilized for each genotypic group.

#### RNA Extraction

Fresh flash frozen brain samples were thawed on ice, and hemispheres were homogenized in 300 μl Qiazol (Qiagen) using a motorized tissue ruptor, topped with 700ul of Qiazol, and mixed. Tubes were incubated at RT for 5 min, and 200 μl chloroform was added to the mixture. Tubes were incubated at RT for 2–3 min, and then centrifuged at 12,000 g for 15 min at 4°C. DNA was removed and RNA was extracted using the RNeasy kit according to the protocol provided (Qiagen). RNA concentration was tested upon completion.

#### c-DNA Preparation

Single stranded c-DNA was generated from 1ng RNA using the High Capacity cDNA Reverse Transcription Kit according to the protocol provided (Applied Biosystems). c-DNA was stored at −20°C until use.

#### q-RT PCR Reaction and Quantification

Reaction mixture was created by combining c-DNA (diluted 1:20) with forward and reverse primers (100nM), iQ SYBR green 2× mix, and H2O. qRT PCR was run in quadruplicate for each sample. Forward and reverse primer sequences for the following targets of interest can be found in Table 3: glyceraldehyde 3-phosphate dehydrogenase (GAPDH), complement component 1q (C1q), complement component 3 (C3), triggering receptor on myeloid cells 2 (TREM2), and tumor necrosis factor alpha (TNFα). q-RT PCR was performed using the 7900 HT Fast Real-Time PCR machine (Life Technologies). Outlier analysis was performed on quadruplicate CT values for each subject, and average CT per subject was generated. ΔCT was calculated as the difference in expression between housekeeping (GAPDH) CT and target CT for each subject. ΔΔCT was calculated as the difference between average wildtype ΔCT and ΔCT of interest for each subject. Fold change was calculated as 2^ΔΔCT^ for each subject. Log_2_ fold change was calculated for each subject. Graphs represent average Log_2_ fold change. ΔCT averages were compared across genotypes using One-Way ANOVA with a Bonferroni *post hoc* test, where *n* = 3 animals/genotype.

**TABLE 3:** Primers utilized in qRT-PCR experiments.

**Primer name**	**Primer sequence**	**Length (bp)**	**NCBI Refseq #**	**Journal reference**
C1qa-1F	CAAGGACTGAAGGGCGTGAA	20	NM_007572.2	
C1qa-1R	CAAGCGTCATTGGGTTCTGC	20		
C3-2F	GACGCCACTATGTCCATCCT	20	NM_009778.3	
C3-2R	CCAGCAGTTCCAGGTCCTTTG	21		
GAPDH_480F	GGCCAAGGTCATCCATGAC	19	NM_008084.3	
GAPDH_562R	CAGTCTTCTGGGTGGCAGTG	20		
TNF-2F	GGGGCCACCACGCTCTTCTGTC	22	NM_000594.4	Font-Nieves et al., 2012
TNF-2R	TGGGCTACGGGCTTGTCACTCG	22		
Trem2-1F	AGCACCTCCAGGCAGGTTTC	20	NM_001272078.1	
Trem2-1R	GATTCCTTGGAAAGAGGAGGAAGG	24		

## Results

### Effects of TIA1 Haploinsufficiency and Knockout on Microgliosis

**FIGURE 1:**
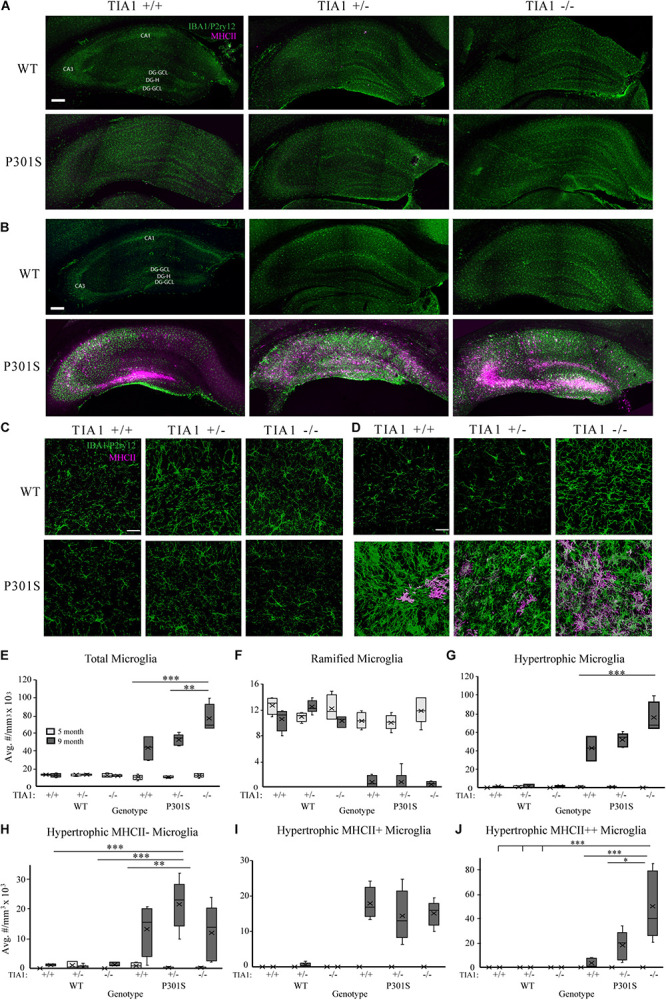
TIA1 knockout exacerbates microgliosis in advanced tauopathy. **(A,B)** Low resolution (10×) fluorescent confocal image stacks depicting the dorsal hippocampus in WT and P301S animals that are TIA1^+/+,^ TIA1^+/–^, and TIA1^–/–^ at 5 months **(A)** and 9 months **(B)**. Microglia (IBA1 and P2ry12) are visualized in green and MHCII complex are visualized in magenta. Scale = 200 μm. Hippocampal regions labeled: Cornu Amonis 1 (CA1), Cornu Amonis 3 (CA3), Dentate Gyrus Hilus (DG-H), Dentate Gyrus Granule Cell Layer (DG-GCL). **(C,D)** High resolution (40×) confocal image stacks depicting the apex and adjacent dentate gyrus including the dorsal and ventral dentate granule cell layers and hilus in WT and P301S animals that are TIA1^+/+^, TIA1^+/–^, and TIA1^–/–^ at 5 months **(C)** and 9 months **(D)**. Microglia (IBA1 and P2ry12) are visualized in green and MHCII complex are visualized in magenta. Scale = 25 μm **(E)** Average density of total microglia in WT and P301S animals that are TIA1^+/+^, TIA1^+/–^, and TIA1^–/–^ at 5 months (light gray) and 9 months (dark gray). **(F)** Average density of total ramified microglia in WT and P301S animals that are TIA1^+/+^, TIA1^+/–^, and TIA1^–/–^ at 5 months (light gray) and 9 months (dark gray). **(G)** Average density of total hypertrophic microglia in WT and P301S animals that are TIA1^+/+^, TIA1^+/–^, and TIA1^–/–^ at 5 months (light gray) and 9 months (dark gray). **(H)** Average total density of MHCII- hypertrophic microglia in WT and P301S animals that are TIA1^+/+^, TIA1^+/–^, and TIA1^–/–^ at 5 months (light gray) and 9 months (dark gray). **(I)** Average density of MHCII + hypertrophic microglia in WT and P301S animals that are TIA1^+/+^, TIA1^+/–^, and TIA1^–/–^ at 5 months (light gray) and 9 months (dark gray). **(J)** Average density of MHCII ++ hypertrophic microglia in WT and P301S animals that are TIA1^+/+^, TIA1^+/–^, and TIA1^–/–^ at 5 months (light gray) and 9 months (dark gray). *n* = 4–7 subjects/genotype for all measures. All statistical comparisons calculated with One-Way ANOVA with Bonferroni *post hoc* test, ^∗^*p* ≤ 0.05, ^∗∗^*p* ≤ 0.01, ^∗∗∗^*p* ≤ 0.001.

Microgliosis occurs in the hippocampus of P301S transgenic mice, with microglial reactivity beginning as early as 4 months of age, and increasing in severity with disease progression (Yoshiyama et al., 2007; López-González et al., 2015; Schweig et al., 2017; Dejanovic et al., 2018; Sayed et al., 2018). We characterized microglial density, morphologic changes, and antigen presentation (MHCII immunoreactivity) in the hippocampus of wildtype or P301S transgenic mice that expressed TIA1^+/+^, TIA1^+/–^, or TIA1^–/–^ at both 5 and 9 months. Patterns of IBA1/P2ry12 and MHCII immunoreactivity were assessed qualitatively across the entire hippocampal formation (Figures 1A,B). At 5 months, there was a higher number of microglial cells in WT TIA1^–/–^, and in each of the P301S groups compared to wildtype. Microglia were predominantly ramified in morphology, with a small fraction exhibiting hypertrophy, indicating a partial transition from a homeostatic to a reactive state. However, no MHCII mediated antigen presentation was observed in any genotypic group at this age (Figure 1A). At 9 months, there was a dramatic increase in number of microglial cells in all P301S groups compared to wildtype, and compared to levels observed at 5 months within the same genotypic group. Microglia in all P301S mice were almost exclusively hypertrophic or amoeboid in morphology, indicating increased reactivity. MHCII labeled cells were strongly localized to CA3 and the dentate gyrus hilus in all P301S groups, however label was widely distributed in the P301S TIA1^+/–^ group compared to P301S TIA1^+/+^ and P301S TIA1^–/–^. P301S TIA1^+/–^ and P301S TIA1^–/–^ subjects exhibited higher MHCII reactivity adjacent to the hippocampal fissure and in CA1 compared to P301S TIA1^+/+^ subjects, indicating that reduced TIA1 expression is associated with increased antigen presentation (Figure 1B).

3D cell counting techniques were used to quantify microglial density, morphologic change, and antigen presentation in the dentate gyrus and hilus. At 5 months, there were no statistically significant differences in total microglial density between genotypic groups (Figures 1C,E). All genotypes demonstrated predominantly ramified microglial morphology (Figures 1C,F,G), however we observed a trend toward a higher number of MHCII-hypertrophic cells in the P301S TIA1^+/+^ group compared to WT TIA1^+/+^ and ^–/–^ (Figures 1C,H; *p* = 0.059 One-Way ANOVA, Bonferroni *post hoc*). These results suggest a preferential priming of microglial cells toward a reactive phenotype in P301S TIA1^+/+^ mice at 5 months compared to P301S TIA1^+/–^ and P301S TIA1^–/–^ subjects. No MHCII reactivity was detected in any group at this time, indicating that microglia have not yet phagocytosed and processed antigen to trigger downstream neuroinflammation (Figures 1C,I,J).

At 9 months, total microglial density was significantly higher in all P301S groups compared to all wildtype groups (all *p* ≤ 0.003, One-Way ANOVA, Bonferroni *post hoc*). Microglial density was also significantly higher compared to those within each P301S genotypic group at 5 months (all *p* ≤ 0.001, Student’s *t*-test) (Figures 1D,E). These results show increased proliferation and/or infiltration of microglial cells with the progressing insult of tauopathy and neurodegeneration from 5 to 9 months. There was a significantly higher density of total microglia in P301S TIA1 ^–/–^ animals compared to both P301S TIA1^+/+^ and P301S TIA1^+/–^ subjects (TIA1^–/–^ vs. TIA1^+/+^ *p* < 0.001; TIA1^–/–^ vs. TIA1^+/–^ *p* = 0.007, One-Way ANOVA, Bonferroni *post hoc*), linking loss of TIA1 to increased neuroimmune activity (Figure 1E). Consistent with these results, there was a significantly lower density of ramified microglia in all P301S groups compared to wildtype (all *p* < 0.001, One-Way ANOVA, Bonferroni *post hoc*) (Figures 1D,F). Furthermore, there was a significantly higher density of hypertrophic microglia in all P301S groups compared to wildtype (all *p* < 0.001, One-Way ANOVA, Bonferroni *post hoc*), and compared to levels observed within genotypic group at 5 months (all *p* < 0.001, Student’s *T*-test) (Figures 1D,G). A shift from primarily ramified morphology to primarily hypertrophic morphology within the population of resident microglia represents a switch from homeostatic to reactive function in the long-term presence of tau pathology. The P301S TIA1^–/–^ group presented with significantly higher density of total hypertrophic cells compared to P301S TIA1^+/+^ and P301S TIA1^+/–^ groups (TIA1^–/–^ vs. TIA1^+/+^ *p* = 0.001; TIA1^–/–^ vs. TIA1^+/–^ *p* = 0.01, One-Way ANOVA, Bonferroni *post hoc*), signifying an exacerbation of microglial reactivity when TIA1 is absent (Figure 1G).

Additionally, at 9 months, the density of MHCII- hypertrophic cells was significantly higher in P301S TIA1^+/–^ subjects compared to wildtype (*p* = 0.001, One-Way ANOVA, Bonferroni *post hoc*), while P301S TIA1^+/+^ and P301S TIA^–/–^ groups did not significantly differ from wildtype (Figure 1H). There were no significant differences in the density of MHCII-cells between P301S groups, and each 9-month-old P301S group had a higher mean density of MHCII- compared to those observed within the same genotypic group at 5 months (TIA1^+/+^ *p* = 0.016, TIA1^+/–^ *p* < 0.001, TIA1^–/–^ *p* = 0.24, Student’s *T*-test) (Figures 1D,H). All 9 month P301S groups exhibited a significantly higher mean density of MHCII + hypertrophic cells compared to wildtype groups (all *p* < 0.001, One-Way ANOVA, Bonferroni *post hoc*), and compared to levels observed within each genotypic group at 5 month (all *p* < 0.001, Student’s *T*-test), with no differences between groups (Figures 1D,I). Importantly, 9 month P301S TIA1 ^–/–^ subjects exhibited significantly higher density of MHCII + + hypertrophic cells compared to all wildtype groups (all *p* < 0.001, One-Way ANOVA, Bonferroni *post hoc*), while other P301S groups did not significantly differ from wildtype (Figures 1D,J). P301S TIA1^–/–^ subjects also presented with significantly higher density of MHCII + + hypertrophic cells than both P301S TIA1^+/+^ and P301S TIA1^+/–^ groups (TIA1^–/–^ vs. TIA1^+/+^ *p* = 0.001, TIA1^–/–^ vs. TIA1^+/–^ *p* = 0.019, One-Way ANOVA, Bonferroni *post hoc*) (Figures 1D,J), consistent with higher levels of microglial phagocytosis and antigen presentation in these subjects. 9 month P301S TIA1^+/–^ and P301S TIA1^–/–^ groups exhibited significantly greater densities of MHCII++ hypertrophic microglia compared to those observed within their genotypic group at 5 months (TIA1^+/–^ *p* = 0.01, TIA1^–/–^ *p* = 0.002) (Figures 1D,J), demonstrating an increase in antigen presentation with progression of disease and reduction of TIA1. No differences were observed between wildtype groups across any conditions, therefore increased microglial reactivity associated with TIA1 reduction occurs only in the presence of a stressor such as the tau pathology in P301S mice (Figures 1D–J). Taken together these results demonstrate that loss of TIA1 leads to increased microglial reactivity in advanced stages of tauopathy.

### Effects of TIA1 Haploinsufficiency and Knockout on Microglial Phagocytosis of Phospho-Tau

**FIGURE 2:**
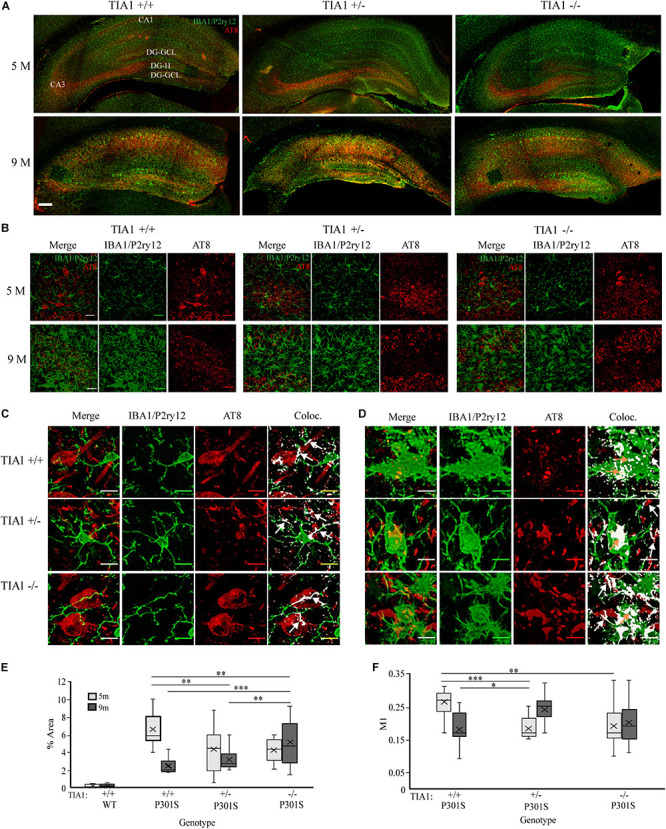
TIA1 reduction increases microglial phagocytosis of phospho-tau in advanced tauopathy. **(A)** Low resolution (10×) fluorescent confocal image stacks depicting the dorsal hippocampus in P301S animals that are TIA1^+/+^, TIA1^+/–^, and TIA1^–/–^ at 5 months (top) and 9 months (Bottom). Microglia (IBA1/P2ry12) are visualized in green and phospho-tau (AT8) is visualized in red. Hippocampal regions labeled: Cornu Amonis 1 (CA1), Cornu Amonis 3 (CA3), Dentate Gyrus Hilus (DG-H), Dentate Gyrus Granule Cell Layer (DG-GCL). Scale = 200 μm **(B)** High resolution (40×) confocal image stacks depicting the apex and adjacent dentate gyrus including the dorsal and ventral dentate granule cell layers and hilus in P301S subjects that are TIA1^+/+^, TIA1^+/–^, and TIA1^–/–^ at 5 months (top) and 9 months (bottom). Microglia (IBA1/P2ry12) are visualized in green and phospho-tau (AT8) is visualized in red. Scale = 25 μm **(C,D)** High resolution (40×) confocal image stacks illustrating colocalization (white mask) of IBA1/P2ry12 (green) and AT8 (red), in subsets of images from **(B)**. Colocalization represents overlap of green and red particles in merged 2D image stack. **(C)** at 5 months, **(D)** at 9 months. Scale = 10 μm. White arrows = apposition of microglia to AT8 + structure, orange arrows = internalization of AT8 + structure by microglia **(E)** Percent area occupied by AT8 particles in **(B)**, 5 months (light gray), 9 months (dark gray). *n* = 12–15 fields from 4 to 5 animals/genotype. **(F)** Manders coefficient 1 (M1) = % overlap of IBA1/P2ry12 particles with AT8 particles in **(B)**. 5 months (light gray), 9 months (dark gray). *n* = 12–15 fields from 4 to 5 animals/genotype. All statistical comparisons calculated with One-Way ANOVA with Bonferroni *post hoc* test, ^∗^*p* ≤ 0.05, ^∗∗^*p* ≤ 0.01, ^∗∗∗^*p* ≤ 0.001.

Expression of the MHCII complex is indicative of an increase in antigen presentation, and is associated with increased phagocytic activity in microglial cells (reviews: Hanisch and Kettenmann, 2007; Lynch, 2009; Schetters et al., 2018; Das and Chinnathambi, 2019). Phospho-tau reactive structures (AT8), all microglial cells (IBA1/P2ry12), and specifically the subpopulation of antigen presenting microglial cells (MHCII) were visualized to estimate phagocytosis of phospho-tau by microglia. First, patterns of AT8 expression were analyzed qualitatively across the hippocampal formation. At 5 months, strong AT8 expression was seen in CA2-3 and in the dentate gyrus in all P301S groups, while at 9 months, widespread AT8 reactivity was present in all parts of the hippocampus in all P301S groups (Figure 2A). MHCII expression followed this progression of AT8 + tau pathology, with robust colocalization of the two markers in hippocampal areas impacted by early phospho-tau load (CA3 and the dentate gyrus hilus), and lower levels of colocalization in areas with advanced phospho-tau accumulation (CA1 and the hippocampal fissure) (Figures 1A,B, 2A). Negligible MHCII and AT8 expression was detected in wildtype controls at both ages (Figures 1A,B, 2A, TIA1 ± and ^–/–^ controls not shown).

Total phospho-tau load in the dentate gyrus was assessed in each group by quantifying percent area occupied by AT8 particles. At 5 months AT8 expression was significantly higher in P301S TIA1^+/+^ subjects compared to all other groups (all *p* ≤ 0.001, One-Way ANOVA, Bonferroni *post hoc*), indicating a potential slowing of phospho-tau spread early in disease progression with TIA1 reduction, similar to results observed in our previous work (Figures 2B,E; Apicco et al., 2017). However, at 9 months AT8 expression was significantly higher in P301S TIA1^–/–^ subjects compared to all other groups (all *p* ≤ 0.002, One-Way ANOVA, Bonferroni *post hoc*), indicating that the absence of TIA1 exacerbates phospho-tau load in this vulnerable region as disease progresses. At 9 months, AT8 expression was significantly lower in P301S TIA1^+/+^ subjects compared to levels observed at 5 months (*p* < 0.001, Student’s *T*-test) (Figures 2B,E).

To determine the relationship between microglia and phospho-tau containing neuronal structures, colocalization of AT8 particles with IBA1/P2ry12 particles and with MHCII particles was quantified. At 5 months, colocalization occurred when microglial processes apposed AT8 + structures (Figure 2C). Percent overlap of IBA1/P2ry12 particles with AT8 particles (Manders’ coefficient 1, M1) was significantly higher in P301S TIA1^+/+^ subjects compared to both P301S TIA1^+/–^ and P301S TIA1^–/–^ subjects (TIA1^+/+^ vs. TIA1^+/–^ *p* < 0.001, TIA1^+/+^ vs. TIA1^–/–^ *p* = 0.002, One-Way ANOVA, Bonferroni *post hoc*) (Figure 2F). Higher overlap in the P301S TIA1^+/+^ group was expected, given the increased level of phospho-tau expression observed (Figures 2B,E), and the nature of microglial interactions with AT8 + structures (Figure 2C). At 9 months, colocalization also occurred when processes apposed AT8 + structures, however there was an increase in internal colocalization of phospho-tau particles with microglial particles, particularly in P301S TIA1^+/–^ subjects (Figure 2D), indicating that phospho-tau is internalized by reactive microglia in these subjects. Percent overlap of particles was significantly higher in P301S TIA1^+/–^ subjects compared to P301S TIA1^+/+^ (*p* = 0.026, One-Way ANOVA, Bonferroni-*post hoc*). P301S TIA1^+/–^ subjects exhibited a significantly higher percent overlap at 9 months compared to values observed at 5 months (*p* < 0.001, Student’s *T*-test), while percent overlap was significantly lower in P301S TIA1^+/+^ subjects at 9 months compared to values observed at 5 months (*p* < 0.001, Student’s *T*-test) (Figure 2F). Qualitatively, we observed AT8 reactive particles were localized within the microglial cytoplasm of MHCII reactive microglia (data not shown), however there were no significant differences in percent overlap of these two markers between P301S groups.

#### Ultrastructural Analysis of Microglia and Phospho-Tau Containing Neuronal Structures

**FIGURE 3:**
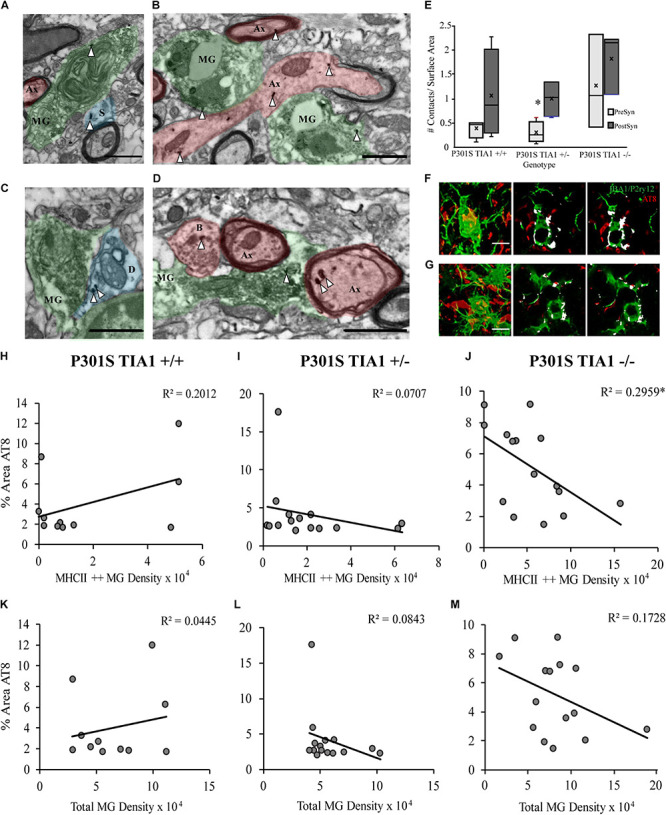
Microglia preferentially interact with pre-and post-synaptic structures containing tau in P301S TIA1^–/–^, while microglia in P301S TIA1 ± interact with post-synaptic structures containing tau in advanced tauopathy. **(A–D)** Electron photomicrograph at 20k × magnification depicting IBA1 + microglia (MG, green) labeled with DAB contacting pre-synaptic (red) and post-synaptic (blue) neuronal structures in dentate gyrus hilus. Scale = 1 μm: **(A)** An IBA1 + microglia co-labeled with AT8 (gold, white arrowhead) contacting an AT8 + dendritic spine (S, blue) and an unlabeled axon (Ax, red) in a P301S TIA1^–/–^ subject. **(B)** An IBA1 + microglia co-labeled with AT8 (gold, white arrowheads) contacting two AT8 + (gold, white arrowheads) axons (Ax, red) in a P301S TIA1^–/–^ subject. **(C)** An IBA1 + microglia contacting an AT8 + (gold, white arrowheads) dendrite (**D**, blue) in a P301S TIA1 ± subject. **(D)** An IBA1 + microglia co-labeled with AT8 (gold, arrow arrowheads) contacting an AT8 + (gold, white arrowheads) bouton (**B**, red) and two AT8 + (gold, arrowheads) axons (Ax, red) in a P301S TIA1^–/–^ subject. **(E)** Density of presynaptic (light gray) and post synaptic (dark gray) AT8 + neuronal contacts on surface area of microglia in P301S TIA1^+/+^, TIA1^+/–^, and TIA1^–/–^ subjects at 9 months (*n* = 3–4 microglia from one animal/genotype). **(F,G)** Serial adjacent optical slices from high-resolution confocal image stacks (in 2B), showing internalization (colocalizatized signal overlap, white) of AT8 labeled structures (red) in microglia (IBA1/P2ry12, green), in a 9 month **(F)** a P301S TIA1^+/–^ subject and; **(G)** a P301S TIA1^–/–^ subject. Scale = 10 μm. **(H–J)** Correlation and regression analyses between percent area occupied by AT8 particles and MHCII + + microglial density in P301S TIA1^+/+^ **(H)**, P301S TIA1^+/–^ **(I)**, and P301S TIA1^–^ **(J)** subjects. **(K–M)** Correlation and regression analyses between percent area occupied by AT8 particles and total microglial density in P301S TIA1^+/+^ **(K)**, P301S TIA1^+/–^ **(L)**, and P301S TIA1^–/–^ **(M)** subjects. All statistical comparisons calculated with One-Way ANOVA with Bonferroni *post hoc* test, Correlations performed using bivariate Person’s Correlation in SPSS, ^∗^*p* ≤ 0.05.

Contents of microglial cells and contacts with neural structures were assessed at the ultrastructural level in the dentate gyrus hilus. Microglial cells containing phagocytosed debris in P301S TIA1^+/–^ and P301S TIA1^–/–^ subjects also contained gold labeled aggregates, supporting light microscopic histologic results (Figures 3A,B,D,F,G). Contacts with all neural structures were quantified, and categorized as pre- or post-synaptic, and phospho-tau positive or negative (Figure 3E). All microglia contacted more post-synaptic tau + structures compared to presynaptic tau + structures. Microglia in the P301S TIA1^–/–^ subject appeared to contact more presynaptic structures compared to other genotypes, however these results did not reach significance because of the high degree of variance (Figures 3B,D,E).

#### Correlations Between Phospho-Tau Expression and Microgliosis

Microgliosis can increase phospho-tau load through the release of pro-inflammatory cytokines, and contribute to the propagation of tau pathology through phagocytosis of phospho-tau (Lee et al., 2010; Sy et al., 2011; Majerova et al., 2014; Asai et al., 2015; Bolós et al., 2015; Luo et al., 2015; Wang et al., 2017; Hopp et al., 2018, reviews: Zilka et al., 2012; Perea et al., 2018). Here, we performed correlation and regression analyses to determine whether phospho-tau expression was related to microglial density and antigen presentation. There was a significant negative correlation between percent area occupied by AT8 particles and density of MHCII ++ microglial cells in P301S TIA1^–/–^ subjects (*p* = 0.036, Pearson’s Correlation, *R*^2^ = 0.296) (Figure 3J). Thus, in TIA1 knockout subjects (but no other group), there are lower levels of phospho-tau expression when there is a higher density of antigen presenting microglia. These results suggest that increased inflammation is not directly related to increased aggregation of insoluble phospho-tau when TIA1 is removed. Additionally, this may indicate that MHCII reactive microglia phagocytose phospho-tau. No other significant correlations were detected between percent area occupied by AT8 particles and total microglial density in any group (Figures 3H,I,K–M). However, a negative correlation between percent area occupied by AT8 particles and total microglial density reached the trend level in TIA1 knockout subjects (*p* = 0.123, Pearson’s Correlation, *R*^2^ = 0.173) (Figure 3M), further supporting the idea that increased microglial reactivity does necessarily lead to increased levels of insoluble phospho-tau aggregation observed in TIA1 knock-out subjects.

### Effects of TIA1 Haploinsufficiency and Knockout on Gene Expression of Microglial Phagocytic Markers

**FIGURE 4:**
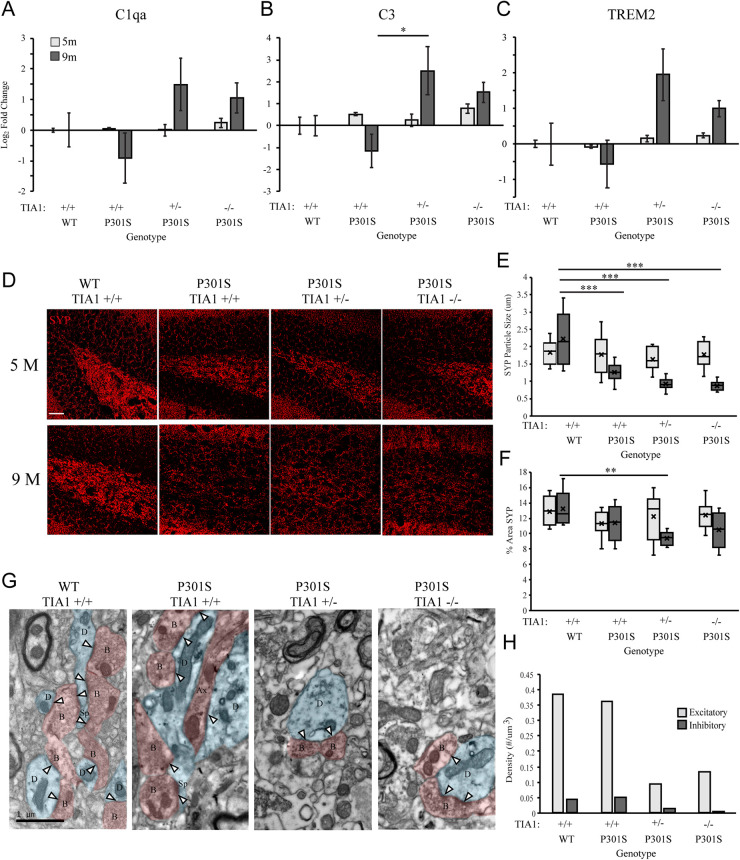
Reduction of TIA1 increases expression of mRNAs associated with microglial phagocytosis and increases synapse loss in advanced tauopathy. **(A)** log2 fold change of C1qa expression in WT TIA1^+/+^ vs. P301S TIA1^+/+^, P301S TIA1^+/–^, and P301S TIA1^–/–^ at 5 months (light gray) and 9 months (dark gray). *n* = 3 subjects/genotype. **(B)** log2 fold change of C3 expression in WT TIA1^+/+^ vs. P301S TIA1^+/+^, P301S TIA1^+/–^, and P301S TIA1^–/–^ at 5 months (light gray) and 9 months (dark gray). *n* = 3 subjects/genotype. **(C)** log2 fold change of TREM2 expression in WT TIA1^+/+^ vs. P301S TIA1^+/+^, P301S TIA1^+/–^, and P301S TIA1^–/–^ at 5 months (light gray) and 9 months (dark gray). *n* = 3 subjects/genotype. **(D)** High resolution (40×) confocal maximum projection images depicting Synaptophysin (SYP, red) in the apex of the dentate gyrus including the dorsal and ventral dentate granule cell layers and hilus in WT TIA1^+/+^ and P301S subjects that are TIA1^+/+^, TIA1^+/–^, and TIA1^–/–^ at 5 months (top) and 9 months (bottom). Scale = 25 μm. **(E)** Average size of SYP particles in **(D)**, 5 months (light gray) and 9 months (dark gray). *n* = 9–12 fields, from 3 to 4 animals/genotype. **(F)** Percent Area occupied by SYP particles in **(D)**, 5 months (light gray) and 9 months (dark gray). *n* = 9–12 fields, from 3 to 4 animals/genotype. **(G)** Electron photomicrographs at 20k x depicting synapses (Presynaptic structures in red, B, bouton; Ax, axon; Postsynaptic structures in blue, D, dendrite; Sp, spine; Synaptic contacts = white arrowheads) in the dentate gyrus hilus neuropil in WT TIA1^+/+^ and P301S subjects that are TIA1^+/+^, TIA1^+/–^, and TIA1^–/–^ animals. Scale = 1 μm. **(H)** Average density of excitatory (light gray) and inhibitory (dark gray) synapses in the dentate gyrus hilus neuropil in WT TIA1^+/+^ and P301S TIA1^+/+^, TIA1^+/–^, and TIA1^–/–^ animals. (*n* = 3–4 fields, from 1 animal/genotype, no statistical analyses performed, qualitative data). All statistical comparisons calculated with One-Way ANOVA with Bonferroni *post hoc* test, ^∗^*p* ≤ 0.05, ^∗∗^*p* ≤ 0.01, ^∗∗∗^*p* ≤ 0.001.

We next directly assessed the effect of TIA1 reduction on levels of microglial phagocytic activity in the P301S mouse through quantification of mRNAs associated with the phagocytic program. Using qRT-PCR, expression of Complement C1q subcomponent subunit alpha (C1qa), Complement C3 subcomponent (C3), and Triggering Receptor Expressed on Myeloid Cells 2 (TREM2) was measured in brain lysates from mice that were wildtype TIA1^+/+^ or P301S expressing TIA1^+/+^, TIA1^+/–^, or TIA1^–/–^ at both 5 and 9 months. At 5 months, mRNA expression levels were comparable to wildtype for all phagocytic markers across all P301S groups (Figures 4A–C). However, at 9 months expression of C3 mRNA was higher in P301S TIA1^+/–^ and P301S TIA1^–/–^ groups compared to P301S TIA1^+/+^ and wildtype, with expression 5.7-fold higher in P310S TIA1^+/–^ subjects, and 2.9-fold higher in P301S TIA1^–/–^ subjects compared to wildtype. P301S TIA1^+/–^ subjects demonstrated significantly higher expression of C3 compared to P301S TIA1 +/+ (*p* = 0.048, One-Way ANOVA, Bonferroni *post hoc*), and P301S TIA1^–/–^ subjects exhibited significantly higher C3 mRNA expression compared to levels observed within the genotypic group at 5 months (*p* = 0.048, Student’s *T*-test) (Figure 4B). C1qa mRNA expression was also higher in P301S TIA1^+/–^ and P301S TIA1^–/–^ groups compared to P301S TIA1^+/+^ and wildtype. While differences did not reach significance, expression of C1qa was 2.8-fold higher in P301S TIA1 ± and 2-fold higher in P310S TIA1^–/–^ compared to wildtype (Figure 4A). These results show increased expression of opsonizing molecules that induce microglial phagocytosis when TIA1 is reduced in tauopathy. At 9 months, there was also higher expression of TREM2 mRNA in P301S TIA1^+/–^ and P301S TIA^1–/–^ groups compared to P301S TIA1^+/+^ and wildtype, with expression 3.9-fold higher in P301S TIA1^+/–^ and 2-fold higher in P301S TIA1^–/–^ subjects compared to wildtype. P301S TIA1^–/–^ subjects exhibited significantly greater TREM2 mRNA expression compared to levels observed at 5 months (*p* = 0.024, Student’s *T*-test) (Figure 4C). These results further support an increase in microglial phagocytosis in late stage tauopathy when TIA1 is reduced.

### Effects of TIA1 Haploinsufficiency and TIA1 Knockout on Synapse Loss

C1q and C3 have been shown to mediate microglial phagocytosis of synapses in the P301S mouse model (Dejanovic et al., 2018; Litvinchuk et al., 2018; Wu et al., 2019). Given our results indicating significantly increased expression of complement component C3 with TIA1 reduction, we next investigated effects of TIA1 haploinsufficiency and TIA1 knockout on synapse loss in tauopathy. First, we assessed expression of the pre-synaptic marker synaptophysin (SYP) in the dentate gyrus, through quantification of SYP particle size, and the percent area occupied by SYP particles in subjects that were WT TIA1^+/+^ and P301S TIA1^+/+^, TIA1^+/–^, and TIA1^–/–^ at both 5 and 9 months (Figure 4D). At 5 months, no significant differences were found between any genotypic groups for either measure (Figures 4D–F). However, at 9 months SYP particle size was significantly lower in all P301S groups compared to wildtype, and compared to those observed within each genotypic group at 5 months (all *p* ≤ 0.001, One-Way ANOVA, Bonferroni *Post hoc*; all *p* ≤ 0.03, Student’s *T*-test), indicating loss of presynaptic terminals in progressive tauopathy in these mice (Figures 4D,E). Additionally, at 9 months there was significantly lower percent area occupied by SYP particles in P301S TIA1^+/–^ subjects compared to wildtype subjects and compared to levels observed within this genotypic group at 5 months (*p* = 0.006, One-Way ANOVA, Bonferroni *post hoc*; *p* = 0.015 Student’s *T*-test) (Figures 4D,F). There was a trend toward a lower percent area occupied by SYP particles in P301S TIA1^–/–^ subjects compared to wildtype (*p* = 0.09, One-Way ANOVA, Bonferroni *post hoc*) (Figures 4D,F). No significant differences were detected between P301S genotypic groups at this age (Figures 4D,F). These results indicate loss of presynaptic terminals from projections entering the dentate gyrus when TIA1 is haploinsuffcient. We further evaluated synapse loss through ultrastructural analysis of synapse density in the dentate gyrus hilus of WTTIA1^+/+^ and P301S TIA1^+/+^, TIA1^+/–^, and TIA1^–/–^ subjects at 9 months. Synapses were categorized as either asymmetric (excitatory), or symmetric (inhibitory), and occurring on either a spine or dendrite (Figure 4G). We observed greater than 70% reduction in density of both excitatory and inhibitory synapses in P301S TIA1 ± and P301S TIA1^–/–^ subjects compared to wildtype (Figures 4G–I). No statistical comparisons were performed due to the use of 1 subject per genotype, these qualitative results are consistent with the statistically significant synaptophysin differences.

### Effects of TIA1 Haploinsufficiency and TIA1 Knockout on Production of Pro-inflammatory Cytokines

**FIGURE 5:**
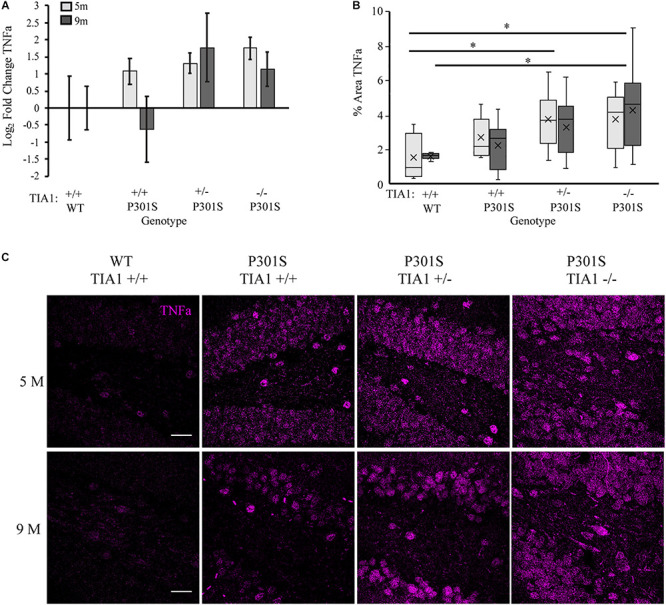
Reduction of TIA1 increases production of TNFα in tauopathy. **(A)** log_2_ fold change of TNFα expression in WT TIA1^+/+^ and P301S subjects that are TIA1^+/+^, TIA1^+/–^, and TIA1^–/–^ at 5 months (light gray) and 9 months (dark gray). *n* = 3 animals/genotype **(B)** Percent area occupied by TNFα particles in **(C)**. 5 months (light gray), 9 months (dark gray). *n* = 9–12 fields, from 3–4 animals/genotype. **(C)** High resolution (40×) confocal image stacks depicting the apex and adjacent dentate gyrus including the dorsal and ventral dentate granule cell layers and hilus in P301S subjects that are TIA1^+/+^, TIA1^+/–^ and TIA1^–/–^ at 5 months (top) and 9 months (bottom). TNFα visualized in purple. Scale = 25 μm. All statistical comparisons calculated with One-Way ANOVA with Bonferroni *post hoc* test, ^∗^*p* ≤ 0.05.

We next assessed how TIA1 haploinsufficiency and TIA1 knockout impact levels of inflammatory cytokines in the P301S mouse brain. First, we quantified TNFα mRNA expression using qRT-PCR in whole-brain lysates of mice that were wildtype TIA1^+/+^ and all P301S groups at both 5 and 9 months. At 5 months, TNFα mRNA expression trended higher in all P301S groups compared to wildtype, with levels higher by 2.1-fold in P301S TIA1^+/+^ mice, 2.5-fold in P301S TIA1^+/–^ mice, and 3.4-fold in P301S TIA1^–/–^ mice. No significant differences were found between P301S groups, however there was an inverse relationship between TIA1 expression and TNFα mRNA expression (Figure 5A). As expected given our previous results, at 9 months, TNFα mRNA expression trended higher in P301S TIA1^+/–^ and P301S TIA1^–/–^ subjects compared to P310S TIA1^+/+^ and wildtype, with levels 3.4-fold higher in P301S TIA1^+/–^ subjects, and 2.2-fold higher in P301S TIA1^–/–^ subjects compared to wildtype (Figure 5A). No significant effect of age was found in regard to TNFα expression (Figure 5A).

Since TIA1 plays a critical role in regulating TNFα mRNA translation during stress, effects of TIA1 haploinsufficiency and TIA1 knockout on TNFα protein expression were determined. Using immunofluorescence and high-resolution laser-scanning confocal microscopy we assessed the anatomical distribution and percent area occupied by TNFα particles in the dentate gyrus. At 5 months, TNFα expression appeared granular and diffuse in the dentate granule cell layers, and was concentrated in hilar mossy cell somata (Figures 5B,C). Percent area occupied by TNFα particles was significantly higher in P301S TIA1^+/–^ (*p* = 0.028, One-Way ANOVA, Bonferroni *post hoc*) and P301S TIA1^–/–^ subjects (*p* = 0.026, One-Way ANOVA, Bonferroni *post hoc*) compared to wildtype (Figure 5B). These results further support qRT-PCR results, and suggest not only increased transcription, but also increased translation of TNFα in these groups compared to P301S TIA1 +/+ and wildtype at 5 months. At 9 months, TNFα expression was seen in both the somata and dendritic processes of dentate granule cells and hilar mossy cells, suggesting neuronal production of TNFα (Figure 5C). Percent area occupied by TNFα particles was significantly higher in only P301S TIA1^–/–^ subjects compared to wildtype (*p* = 0.015, One-Way ANOVA, Bonferroni *post hoc*). However, there was an inverse relationship between TIA1 and TNFα protein expression. No significant differences were observed between protein expression levels at 5 months and 9 months in any group (Figure 5B). Additionally, no differences were observed between wildtype controls at either time point, suggesting that increased TNFα expression relies on the presence of a chronic stressor like tauopathy (data not shown).

**FIGURE 6:**
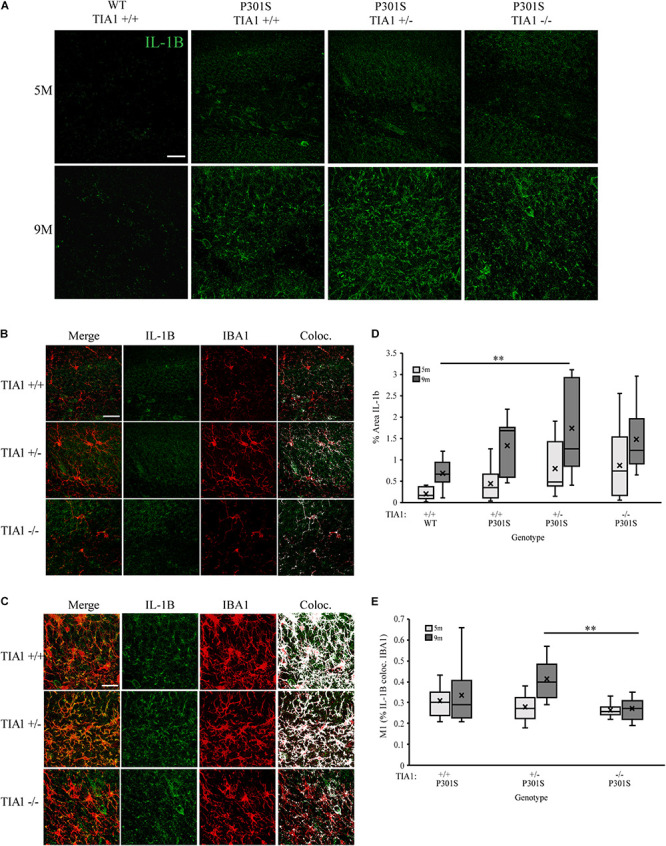
Haploinsufficiency of TIA1 increases IL-1β in advanced tauopathy. **(A)** High resolution (40×) confocal image stacks depicting the apex of the dentate gyrus including the dorsal and ventral dentate granule cell layers and hilus in WT TIA1^+/+^ and P301S subjects that are TIA1^+/+^, TIA1^+/–^, and TIA1^–/–^ at 5 months (top) and 9 months (bottom). IL-1β is visualized in green. Scale = 25 μm. **(B,C)** High resolution (40×) confocal image stacks illustrating colocalization (white) of IL-1β (green) and IBA1 (red), in subsets of images from **(A)**. **(B)** = 5 months, **(C)** = 9 months. Scale = 25 μm. **(D)** Percent area occupied by IL-1β particles in **(A)**. 5 months (light gray), 9 months (dark gray). *n* = 12 fields, from 3 animals/genotype. **(E)** M1 = % IL-1β particles colocalized with IBA1 particles. 5 months (light gray), 9 months (dark gray). *n* = 12 fields, from 3 animals/genotype. All statistical comparisons calculated with One-Way ANOVA with Bonferroni *post hoc* test, ^∗∗^*p* ≤ 0.01.

Expression of pro-inflammatory cytokine IL-1β is known to be regulated by RBP tristetraprolin (TTP) during periods of stress, however recent studies have also implicated TIA1 in regulation of its expression as well (Ash et al., 2014; Carpenter et al., 2014; Haneklaus et al., 2017; Battu et al., 2018; Rayman et al., 2019). Using immunofluorescence and high-resolution laser-scanning confocal microscopy we analyzed the anatomical distribution and percent area occupied by IL-1β particles in the dentate gyrus. At 5 months, IL-1β distribution was diffuse and granular, localized mainly to hippocampal neuronal structures (Figures 6A,B). No significant difference in percent area occupied by IL-1β particles was detected between genotypic groups, however P301S TIA1^+/–^ subjects trended toward a higher percent area occupied compared to wildtype subjects (*p* = 0.109, One-Way ANOVA, Bonferroni *post hoc*) (Figure 6D). At 9 months, IL-1β particles were granular and localized to both neuronal and glial structures, particularly in P301S TIA1^+/–^ subjects (Figures 6A,C). Percent area occupied by IL-1β particles was significantly higher in P301S TIA1^+/–^ subjects compared to wildtype (*p* = 0.010, One-Way ANOVA, Bonferroni *post hoc*), and trended toward a higher level in P301S TIA1^–/–^ subjects compared to wildtype (*p* = 0.107, One-Way ANOVA, Bonferroni *post hoc*) (Figure 6D). Percent area occupied by IL-1β particles was significantly increased at 9 months compared to 5 months in all genotypic groups except P301S TIA1^–/–^ subjects (all *p* ≤ 0.013, Student’s *T*-test) (Figure 6D.) To semi-quantitatively determine the proportion of IL-1β produced in microglial cells specifically, we assessed percent overlap of IL-1β particles with IBA1/P2ry12 particles. While no significant differences were detected at 5 months (Figures 6B,E), at 9 months there was a significantly higher percent overlap of particles in P301S TIA1^+/–^ subjects compared to P301S TIA1^–/–^ subjects, and compared to levels observed within this genotypic group at 5 months (Figures 6C,E). These results further indicate an increase in pro-inflammatory cytokine production with TIA1 haploinsufficiency at 9 months, and provide further evidence of the role of TIA1 in modulating the expression of IL-1β.

### Effects of TIA1 Haploinsufficiency and TIA1 Knockout on Oxidative Stress

**FIGURE 7:**
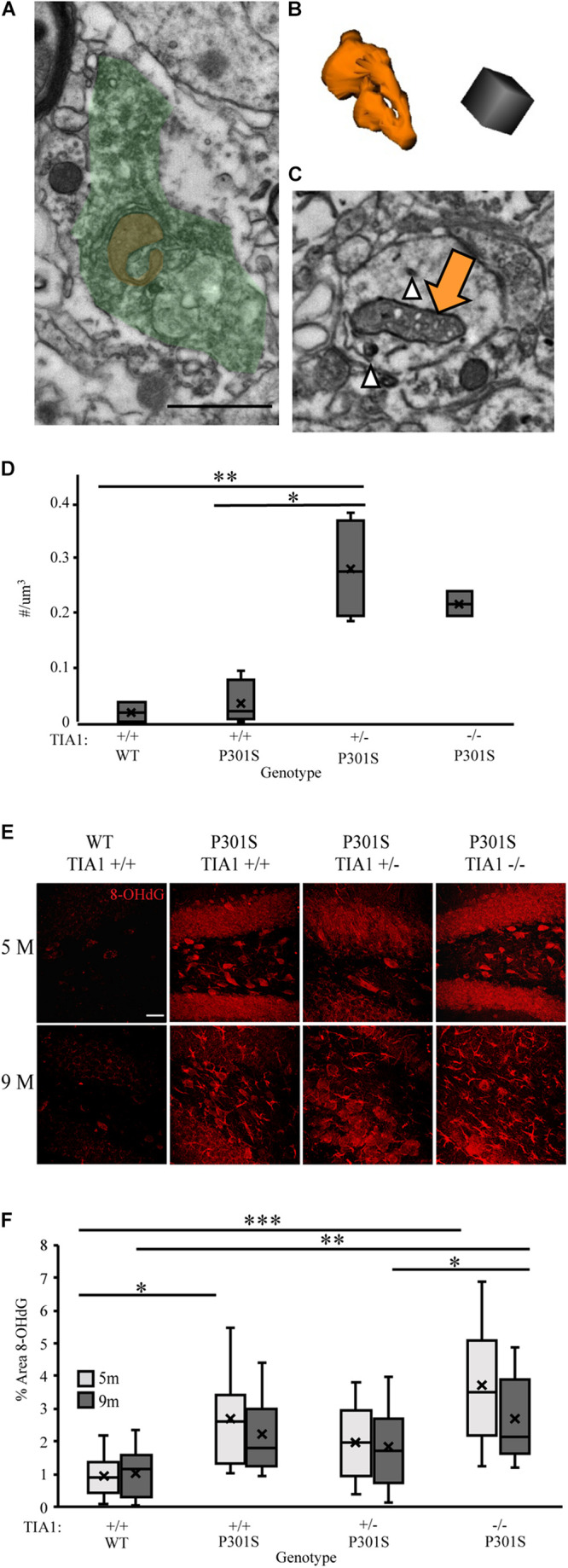
TIA1 reduction increases oxidative stress in tauopathy. **(A)** Electron photomicrograph at 20k × depicting Microglia stained with IBA1 (DAB, green) containing a donut shaped mitochondria (orange) in dentate gyrus hilus of P301S TIA1^–/–^ subject. Scale = 1 μm **(B)** 3D reconstruction of donut mitochondria in **(A)**. Scale = 0.5 μm. **(C)** Electron photomicrograph at 20k x depicting dendrite stained with AT8 (gold particles, white arrowhead) containing a frothy mitochondrion (orange arrow) in dentate gyrus hilus of P301S TIA1^+/–^ subject. Scale = 1 μm **(D)** Density of abnormal mitochondria in WT TIA1^+/+^ and P301S TIA1^+/+^, TIA1^+/–^ and TIA1^–/–^. **(E)** High resolution (40×) confocal image stacks depicting the dentate gyrus adjacent to the apex, including the dorsal and ventral dentate granule cell layers and hilus in P301S subjects that are TIA1^+/+^, TIA1^+/–^, and TIA1^–/–^ at 5 months (top) and 9 months (bottom). 8-OHdG is visualized in red. Scale = 25 μm. **(F)** Percent area occupied by 8-OHdG particles in **(E)**. 5 months (light gray), 9 months (dark gray). *n* = 12 fields, from 3 animals/genotype. All statistical comparisons calculated with One-Way ANOVA with Bonferroni *post hoc* test, ^∗^*p* ≤ 0.05, ^∗∗^*p* ≤ 0.01, ^∗∗∗^*p* ≤ 0.001.

Microglial reactivity and pro-inflammatory cytokine release increase production of ROS, leading to oxidative stress in both microglia and neurons (reviews: Block et al., 2007; Liu et al., 2017). We have demonstrated increased microglial reactivity and inflammatory cytokine production with TIA1 reduction in the P301S mouse, therefore in our next set of experiments we determined its effects on levels of oxidative stress. Specific changes in mitochondrial morphology have been linked to mitochondrial dysfunction and oxidative stress in tauopathies (Yoshiyama et al., 2007; Kopeikina et al., 2011; Duboff et al., 2012; Ahmad et al., 2013; Hara et al., 2014; Long et al., 2015). The density of mitochondria with dystrophic morphology was assessed by carrying out serial immuno-electron microscopy and 3D analysis in the dentate gyrus hilus at 9 months. Abnormal mitochondria that appeared donut shaped/curved (Figures 7A,B), or frothy, containing gaps in the cristae with breakdown of the outer membrane (Figure 7C), were higher in P301S TIA1^+/–^ and P301S TIA1^–/–^ subjects. P301S TIA1^+/–^ subjects had a significantly increased density of abnormal mitochondria compared to both P301S TIA1^+/+^ (*p* = 0.003, One-Way ANOVA, Bonferroni *post hoc*) and wildtype (*p* = 0.008, One-Way ANOVA, Bonferroni *post hoc*) groups (Figure 7D).

To directly assess oxidative stress levels in the hippocampus we assessed expression of 8-Oxo-2’-deoxyguanosine (8-OHdG), a product of oxidative DNA damage resulting from oxidative stress. Using immunofluorescence and high-resolution laser-scanning confocal microscopy we analyzed the anatomical distribution and percent area occupied by 8-OHdG particles in the dentate gyrus. At 5 months, 8-OHdG was highly localized to dentate granule cells and hilar mossy cells in all P301S subjects (Figure 7E). Percent area occupied by 8-OHdG particles was significantly higher in P301S TIA1^+/+^ (*p* = 0.035, One-Way ANOVA, Bonferroni *post hoc*) and P301S TIA1^–/–^ (*p* < 0.001, One-Way ANOVA, Boneferroni *post hoc*) subjects compared to wildtype subjects (Figure 7F). Interestingly, percent area occupied by 8-OHdG was significantly higher in P301S TIA1^–/–^ subjects compared to P301S TIA1^+/–^ subjects (*p* = 0.045, One-Way ANOVA, Bonferroni *post hoc*), consistent with previous data that indicate a protective effect of TIA1 haploinsufficiency early in disease progression (Apicco et al., 2017; Figure 7F). At 9 months, 8-OHdG was localized to neuronal structures as well as glial structures in all P301S groups (Figure 7E). Percent area occupied by 8OHdG particles was significantly higher in only P301S TIA1^–/–^ subjects compared to wildtype (*p* = 0.006, One-Way ANOVA, Bonferroni *post hoc*) (Figure 7F). There was no effect of age on 8-OHdG expression (Figure 7F). These results indicate increased oxidative damage in both early and late stages of tauopathy when TIA1 is knocked out.

## Discussion

Current evidence points toward an active role for neuroinflammation in the progression of tauopathy (reviews: Barron et al., 2017; Leyns and Holtzman, 2017; Salter and Stevens, 2017; Laurent et al., 2018). TIA1 also plays an important role in both peripheral inflammation (Piecyk et al., 2000; Cok et al., 2003; Dixon et al., 2003; Phillips et al., 2004; Kim et al., 2005; Karalok et al., 2014) and in tauopathy (Vanderweyde et al., 2016; Apicco et al., 2017; Maziuk et al., 2018; Jiang et al., 2019), but the involvement of TIA1 in CNS neuroinflammation is poorly understood (Gong and Hewett, 2018; Rayman et al., 2019). Here we demonstrate that, as in the peripheral immune system, TIA1 plays an important role in the neuroimmune response to chronic stress in the form of tauopathy. Thus, reduction or elimination of TIA1 results in significantly increased microgliosis, phagocytosis, and expression of pro-inflammatory cytokines and markers of oxidative stress. These effects were observed only in advanced stage disease, with the exception of TNFα release which was seen both early and late, suggesting that the exacerbation of neuroinflammation by TIA1 reduction occurs following chronic, prolonged stress.

### TIA1 Knockout Exacerbates Microgliosis in Advanced Tauopathy

Tauopathy is marked by infiltration and proliferation of reactive microglial cells in brain regions containing phospho-tau aggregates (Bellucci et al., 2004; Yoshiyama et al., 2007; Sasaki et al., 2008; Bellucci et al., 2011; Maphis et al., 2015). In the P301S mouse model of tauopathy, microgliosis begins at four months and increases progressively with age in the hippocampus, the anatomical region with the highest burden of tau pathology (Bellucci et al., 2004; Yoshiyama et al., 2007; López-González et al., 2015). Here, we showed that knockout of TIA1 in 9-month old P301S mice led to a significant increase in the mean density of reactive microglia in the dentate gyrus relative to control P301S and TIA1 haploinsufficient P301S mice. This increase in microglial number resulting from TIA1 knockout could be due to infiltration of circulating peripheral monocytes that have differentiated, and/or to migration and proliferation of local microglial cells (Wlodarczyk et al., 2014, reviews: Mosher and Wyss-Coray, 2014; Laurent et al., 2018; Li and Barres, 2018; Perea et al., 2018). Pro-inflammatory cytokine TNFα can induce release of CCL2, a chemokine that attracts circulating monocytes into the parenchyma for differentiation (D’Mello et al., 2009; Zhou et al., 2011; Ahmad et al., 2018, review: Laurent et al., 2018). Here we show higher levels of TNFα production in P301S animals with TIA1 reduction, suggesting that there could be increased infiltration of circulating peripheral immune cells in P301S TIA1^–/–^ mice. TNFα can also indirectly increase local microglial proliferation, by increasing astrocytic release of GM-CSF (Selmaj et al., 1990; Lee et al., 1994; Chen et al., 2016). While low-levels of proliferating microglia have been observed in the hippocampus of P301S mice (Leyns et al., 2017), evidence shows that TIA1 regulates cell-cycle protein translation, with TIA1 reduction increasing cellular proliferation *in vitro* (Reyes et al., 2009; Sánchez-Jiménez and Izquierdo, 2015). Knockout of TIA1 in neural tissue has been shown to increase expression of CCNF and CDKN1 (Heck et al., 2014), cell cyclin proteins that are expressed during microglial proliferation after ischemic stroke (McDonough et al., 2020). Knockout of TIA1 may not only impact the inflammatory environment, contributing to microglial proliferation, but it may also increase expression of proteins integral for microglial proliferation. Further studies are required to determine the proportion of differentiated monocytes and proliferative microglial cells in P301S TIA1^–/–^ mice.

### Antigen-Presenting Microglia Are Increased in TIA1 Knockout P301S Mice

Microglia are the resident antigen presenting cells of the CNS, and when chronically activated they phagocytose, process, and present peptide antigens to infiltrating T-lymphocytes via the MHCII complex (reviews: Wyss-Coray and Mucke, 2002; Akiyama et al., 2006; Lynch, 2009; Das and Chinnathambi, 2019). The brains of both human subjects and mouse models of AD exhibit clusters of MHCII presenting reactive microglia in regions of high phospho-tau pathology (McGeer et al., 1988; Perlmutter et al., 1992; Yoshiyama et al., 2007; Romero-Molina et al., 2018, review: Das and Chinnathambi, 2019). For example, in the P301S mouse, MHCII is present in hippocampal microglia and increases in sync with neuropathological progression (Yoshiyama et al., 2007; Romero-Molina et al., 2018). Similarly, we demonstrate that MHCII presenting reactive microglia localize to hippocampal regions most affected by long-term phospho-tau pathology. Microglia phagocytose phospho-tau, which can then be processed and loaded into the MHCII complex for presentation (Majerova et al., 2014; Asai et al., 2015; Bolós et al., 2015; Luo et al., 2015; Maphis et al., 2015, review: Perea et al., 2018). In the present study, we found a significant increase in density of MHCII reactive microglia when TIA1 is knocked out. MHCII antigen presenting cells interact with CD4^+^ T-cells, infiltrating from the periphery (Wyss-Coray and Mucke, 2002). One recent study has described a highly phagocytic MHCII presenting microglial cell, induced by CD4^+^ T-cells reactive to amyloid-β in the 5×FAD model of AD (Mittal et al., 2019). It is possible that in the TIA1 knockout mouse, phospho-tau reactive CD4^+^ T-cells induce a similar type of microglial phenotype. Future studies should focus on characterizing T-cell infiltration, and isolating antigen presented in the MHCII receptor in TIA1 knockout subjects. Overall, these results are consistent with the idea that at normal levels TIA1 decreases phagocytosis and processing of antigen, resulting in reduced adaptive immune functions in tauopathy.

### TIA1 Reduction Increases Microglial Phagocytosis in Advanced Tauopathy

While little is known about stress granule biology in microglia, TIA1 reactive stress granules exist in microglial cells in both human subjects and mouse models of AD (Ghosh and Geahlen, 2015; Vanderweyde, 2015). Ghosh and Geahlen reported that stress granule formation inhibits microglial phagocytic capabilities *in vitro*, and that IgG treatment restored this loss of function (Ghosh and Geahlen, 2015). Our data support the notion that stress granule formation suppresses microglial phagocytosis, since reduction of TIA1 resulted in increased markers of microglial phagocytosis in the brains of P301S mice. In the 5-month old cohort, microglial cells appose to but do not internalize AT8 + neuronal structures, while in the 9-month old cohort many AT8 + puncta were present within the microglial cytoplasm. In advanced stage mice, there is a significantly higher level of phospho-tau internalization by microglia in the dentate gyrus of TIA1 haploinsufficient P301S subjects compared to control P301S subjects. The downstream effects of phospho-tau phagocytosis has not yet been clarified, and it is not known whether this process reduces or paradoxically promotes pathogenesis (Mandrekar et al., 2009; Majerova et al., 2014; Bolós et al., 2015; Chen et al., 2016; Keren-Shaul et al., 2017, reviews: Barron et al., 2017). Indeed, we and others have shown that microglia can package phagocytosed phospho-tau into exosomes, which are then released into the extracellular matrix, further propagating pathology (Asai et al., 2014; Maphis et al., 2015; Hopp et al., 2018).

Recent studies have shown that neuroinflammation, specifically release of inflammatory cytokines, increases tau phosphorylation and aggregation (Li et al., 2003; Kitazawa et al., 2005; Janelsins et al., 2008; Gorlovoy et al., 2009; Garwood et al., 2010; Lee et al., 2010; Maphis et al., 2015; Roe et al., 2011; Sy et al., 2011). We observed both increased levels of neuroinflammation and increased AT8 + phospho-tau aggregation in the dentate gyrus of P301S TIA1 knockout mice compared to other groups. Interestingly, there was a negative correlation between phospho-tau expression and density of antigen presenting microglia in this cohort. This was unexpected given robust evidence in the literature showing that inflammation increases aggregation of insoluble phospho-tau (Li et al., 2003; Kitazawa et al., 2005; Janelsins et al., 2008; Gorlovoy et al., 2009; Garwood et al., 2010; Lee et al., 2010; Maphis et al., 2015; Roe et al., 2011; Sy et al., 2011). Here, we provide several lines of evidence that suggest that TIA1 knockout increases the phagocytic capabilities of microglia. We propose that phospho-tau expression is lowest in environments with the highest density of antigen presenting microglia, due to increased phagocytosis and clearance of phospho-tau.

### Complement Is Increased by Reduction of TIA1

Here, we showed increased expression of C1q and C3 in whole brain lysates of TIA1 haploinsufficient and TIA1 knockout P301S subjects compared to control P301S and wildtype subjects in advanced disease. These results suggest that increased phagocytosis and antigen presentation observed with TIA1 reduction could be linked to increased opsonization of phospho-tau laden structures by complement. In neurodegenerative disease, complement components play an important role in the initiation of phagocytosis by opsonizing pathological structures (Stevens et al., 2007; Schafer et al., 2012; Hong et al., 2016; Vasek et al., 2016; Dejanovic et al., 2018; Litvinchuk et al., 2018, reviews: Brown and Neher, 2014; Salter and Stevens, 2017; Davies and Spires-Jones, 2018; Wilton et al., 2019). In tauopathy the complement factors C1q and C3 are increased in the neural parenchyma and CSF of human AD subjects and in the neural parenchyma of mouse models of AD (Wang et al., 2011; Daborg et al., 2012; Hong et al., 2016; Dejanovic et al., 2018; Litvinchuk et al., 2018; Wu et al., 2019). C1q localizes to synapses, increasing their removal by phagocytic microglia (Dejanovic et al., 2018). Inhibition of both C1q and C3/C3r in mouse models of tauopathy have led to a decrease in microglial phagocytosis of synapses, rescuing neurodegeneration and downstream cognitive deficits (Hong et al., 2016; Dejanovic et al., 2018; Litvinchuk et al., 2018; Wu et al., 2019). These results are consistent with a recent study implicating TIA1 in the regulation of C3 expression (Rayman et al., 2019), however this is the first report of increased complement production with reduction of TIA1 under conditions of stress.

### TREM2 Is Increased by Reduction of TIA1

We observed increased expression of TREM2 in whole brain lysates of P301S subjects with both TIA1 haploinsufficiency and TIA1 knockout compared to P301S and wildtype subjects at 9 months. TREM2 is a myeloid cell receptor involved in activation of the microglial phagocytosis program (Kleinberger et al., 2014; Keren-Shaul et al., 2017, reviews: Brown and Neher, 2014; Deczkowska et al., 2018). Several mutations in the TREM2 gene are risk factors for development of neurodegenerative diseases, including AD (Guerreiro et al., 2013; Jonsson et al., 2013; Sasaki et al., 2015, review: Hickman and El Khoury, 2008). Upregulation of TREM2 expression is a key signature of disease associated microglia (DAMs), and there is evidence that these cells contribute to clearance of amyloid-β pathology in mouse models of AD. However, the function of TREM2 in the context of tauopathy remains a matter for debate (Frank et al., 2008; Wang et al., 2015; Yeh et al., 2016; Keren-Shaul et al., 2017). Studies in the P301S mouse have reported increased pathology with TREM2 deficiency due to ineffectual microglial phagocytic function, and decreased pathology with TREM2 overexpression due to a decrease in neuroinflammation and tau kinase activity, while others have reported a decrease in pathology and neuroinflammation with TREM2 deficiency (Jiang et al., 2016; Leyns et al., 2017; Sayed et al., 2018). Our results are in agreement with the study by Leyns and coworkers (Leyns et al., 2017), which suggested that increased TREM2 expression may exacerbate tauopathy, as we observed increased levels of AT8 + phospho-tau in the dentate gyrus of TIA1 knockout P301S animals.

### TIA1 Reduction Increases Synapse Loss in Advanced Tauopathy

We have recently demonstrated that the RNA-binding protein TIA1 acts as a key modulator of pathophysiology in tauopathies; specifically, reduction of TIA1 ameliorates tau related neurodegenerative processes (Vanderweyde et al., 2016; Apicco et al., 2017; Maziuk et al., 2018; Jiang et al., 2019; reviews: Ash et al., 2014; Wolozin and Ivanov, 2019). A major hallmark of tauopathy is the degeneration and loss of synapses. Here we show increased synapse loss in the dentate gyrus of both TIA1 haploinsufficient and TIA1 knockout mice in advanced tauopathy. These observations are in agreement with results indicating increased microglial phagocytosis, suggesting that this may be targeted specifically to neuronal synapses which have been opsonized for removal. Specific analysis of microglial contents is needed to determine whether TIA1 reduction leads to increased internalization of synaptic proteins. While previous studies in the P301S mouse, including our own, have shown hippocampal synapse loss in early stages of disease (Yoshiyama et al., 2007; Xu et al., 2014; Apicco et al., 2017), we did not observe significant synapse loss in the dentate gyrus of any P301S mice at 5 months. Due to this variability in the P301S mutant phenotype, we did not replicate previous results showing a rescue of synaptophysin expression by TIA1 haploinsufficiency in early stage disease (Apicco et al., 2017). While we have previously demonstrated that TIA1 plays a key role in regulating phospho-tau aggregation dynamics (Vanderweyde et al., 2016; Apicco et al., 2017; Maziuk et al., 2018; Jiang et al., 2019), our observation of increased synapse loss with TIA1 reduction in advanced disease indicates that reducing TIA1 systemically may not always combat neurodegeneration. This discrepancy could be due to heterogeneity in the kinetics of phospho-tau production in P301S mice (Woerman et al., 2017), or due to imbalance between beneficial effects of TIA1 reduction in neurons vs. deleterious effects of TIA1 reduction in the neuroimmune system. This question can best be addressed in an inducible conditional knockout model in which TIA1 is removed specifically in neurons or in microglia of P301S transgenic mice. Studies in this model will provide insight on whether removal of TIA1 in a specific cell-type is more effective at reducing tau related neurodegeneration compared to non-specific TIA1 reduction. Studies in these mice will also help clarify whether exacerbation of inflammation with TIA1 reduction is due to dysregulation of microglial physiology, or to damage related signaling from phospho-tau positive neurons. Additionally, it is possible that the timing of TIA1 removal is of critical importance. While we did not observe significant degeneration of synapses in early stages, we did observe decreased levels of AT8 + tau expression with TIA1 reduction in early stages. Therefore, an inducible conditional model will enable the determination of whether removal at a specific time point is more beneficial for the progression of pathology.

### TIA1 Reduction Increases Production of Pro-inflammatory Cytokines in Tauopathy

The observation of increased reactive microgliosis, antigen presentation, and phagocytosis with TIA1 reduction led to the question of whether TIA1 reduction results in an overall pro-inflammatory environment. TIA1 regulates peripheral expression of inflammatory cytokines like TNFα and IL6 during periods of stress through binding and sequestering the mRNAs for these molecules in stress granules (Piecyk et al., 2000; Phillips et al., 2004; Kim et al., 2005; Lopez de Silanes et al., 2005; Sánchez-Jiménez and Izquierdo, 2015). Studies *in vitro* and in wildtype animals show increased levels of TNFα, Il-6 and COX2 with TIA1 knockout, particularly during cellular stress, however, all experiments were conducted in peripheral organ tissues and systems (Piecyk et al., 2000; Dixon et al., 2003; Phillips et al., 2004; Karalok et al., 2014). The role of TIA1 in regulation of inflammatory cytokines in the central nervous system is poorly understood. In one of the only studies to address this question, Rayman and coworkers reported that overexpression of TIA1 in hippocampal neurons leads to significant increases in expression of pro-inflammatory cytokines, including TNFα, IL-6, and IL-1β (Rayman et al., 2019). We found an increased expression of TNFα mRNA and TNFα protein in P301S TIA1 haploinsufficient and in TIA1 knockout mice compared to control P301S and wildtype mice in early and advanced disease. We also observed increased IL-1β protein expression in P301S TIA1 haploinsufficinet mice in early and advanced disease. Importantly, TIA1 reduction in wildtype mice did not result in increased levels of proinflammatory cytokine expression, indicating that a chronic stressor (e.g., tauopathy) must be present to trigger significant pro-inflammatory cytokine release (data not shown). Taken together, our findings are consistent with those from previous studies characterizing the role of TIA1 in peripheral inflammation, demonstrating a similar role for TIA1 in central neuroinflammation. It follows that under conditions of chronic stress TIA1 serves a protective anti-inflammatory role in the central nervous system. We postulate that removal of TIA1, and decreased incidence of stress granule nucleation, leads to unchecked translation of these harmful pro-inflammatory proteins during cellular stress. When tauopathy is the stressor, increased release of proinflammatory cytokines, likely leads to exacerbation of disease, as these mediators have been shown to increase phosphorylation of tau (Li et al., 2003; Kitazawa et al., 2005; Gorlovoy et al., 2009; Lee et al., 2010; Sy et al., 2011; Zilka et al., 2012). This idea is consistent with our observation of significantly higher AT8 phospho-tau load in advanced stage P301S TIA1 ^–/–^ mice.

### TIA1 Reduction Increases Oxidative Stress in Tauopathy

Activated microglia and damaged neurons release ROS leading to mitochondrial dysfunction and oxidative stress in neurodegenerative diseases, including AD (Block and Hong, 2005; Leszek et al., 2016; Liu et al., 2017). Oxidative stress is an underlying driver of pathology in both human subjects and animal models of AD, with increased ROS levels correlating with increased phospho-tau load and neuronal loss (Good et al., 1996; Takeda et al., 1998; Markesbery and Carney, 1999; Markesbery, 1999; Dumont et al., 2011; Duboff et al., 2012). Abnormal mitochondrial morphologies (Yoshiyama et al., 2007) and abnormal expression of enzymes involved in ROS production (Dumont et al., 2011) have been identified in the brains of P301S mice in mid-stage of disease. We found a similar increase in the density of mitochondria with abnormal frothy and donut shaped morphology in the dentate hilus neuropil of TIA1 haploinsufficient and TIA1 knockout P301S subjects compared to control P301S and wildtype subjects at 9 months. These abnormal mitochondrial profiles can form due to dysregulation of mitochondrial fission and fusion dynamics, and have previously been identified in high oxidative stress environments (Yoshiyama et al., 2007; Kopeikina et al., 2011; Liu and Hajnóczky, 2011; Duboff et al., 2012; Ahmad et al., 2013; Hara et al., 2014; Long et al., 2015). Two recent studies have shown that peripherally, TIA1 controls the translation of mitochondrial internal and external membrane proteins OPA1 and MFF (Carrascoso et al., 2017; Tak et al., 2017). The disordered mitochondrial fusion and abnormal cristae morphology observed here could be due to unchecked translation of these proteins when TIA1 is reduced. We also found increased levels of DNA damage resulting from oxidative stress, with increased expression of oxidative DNA damage marker 8-OHdG in TIA1 knockout P301S subjects at both early and advanced disease states. Oxidative stress is caused by cytochrome-c mediated production of ROS. One study has shown that TIA1 regulates the translation of cytochrome-c *in vitro*, with TIA1 knockout resulting in increased expression (Kawai et al., 2006). Unchecked translation of cytochrome-c could be responsible for increase in ROS production and oxidative stress with observed with TIA1 reduction in the P301S mouse.

### Caveats and Limitations

#### Differences in the Inflammatory Response of TIA1 Haploinsufficient and TIA1 Knockout P301S Mice

Neuroinflammation did not differ between P301S mice with TIA1 haploinsufficiency and P301S mice with TIA1 knockout, with one exception. Microgliosis, including microglial density and antigen presentation, was significantly greater in TIA1 knockout mice compared to TIA1 haploinsufficient mice. These results suggest that there is increased microglial proliferation, or infiltration of peripheral differentiated monocytes in TIA1 knockout subjects, which may be due to unregulated cell cycle progression in the absence of TIA1 (Heck et al., 2014), or increased release of CCL2 (D’Mello et al., 2009; Zhou et al., 2011; Ahmad et al., 2018, review: Laurent et al., 2018). It is possible that increased antigen presentation is due to phagocytosis of phospho-tau oligomers in TIA1 knock-out subjects (Das et al., 2020). Increased antigen presentation differentially affects downstream inflammation and neurodegeneration, as antigens are presented to infiltrating T-cells, which then may carry-out effector functions, fine-tuning the inflammatory response (Wyss-Coray and Mucke, 2002).

#### Sex Balance

In IHC experiments, the 5-month cohort was composed of both male and female subjects. The P301S TIA1^–/–^ group contained 6 males, and the P301S TIA1^+/+^ group contained 4 females and 1 male. In the P301S TIA1^+/–^ group, there were 3 males and 4 females, allowing for statistical comparisons between sexes. We tested for effect of sex on total microglial density, reactive microglial density, and percent area occupied by AT8, TNFα, IL1β, and 8-OHdG particles. Only percent area occupied by 8-OHdG particles exhibited a significant difference between males and females, with females demonstrating higher expression of 8-OHdG (*p* = 0.013, Student’s *T*-test, data not shown). Thus, data from both male and female subjects were pooled for analyses at 5-months. Since it has recently been demonstrated that pathology is present more consistently in P301S-PS19 males compared to females, particularly in advanced disease (Woerman et al., 2017; Sun et al., 2020) all male P301S subjects were studied in the 9-month cohort. Additionally, microglia are responsive to sex hormones (Kodama et al., 2020; review: Kodama and Gan, 2019). Specific miRNAs are differentially active in males compared to females in response to a phospho-tau insult, and when miRNA processing enzyme Dicer is removed, microglia in male -but not female- mice become highly phagocytic (Kodama et al., 2020). Interestingly, sex chromosome gene expression profiles in these subjects are inverse of profiles observed in TIA1 haploinsufficient wildtype mice (Apicco et al., 2019), suggesting that TIA1 expression may fundamentally alter microglial reactivity in a sex specific manner. While not within the scope of the current study, it will be important in the future to measure expression of sex specific genes when TIA1 is reduced in male vs. female P301S subjects, and compare patterns of microglial reactivity between sexes.

#### Environmental Impact

Wildtype mice with TIA1 reduction are known to exhibit peripheral hypersensitive inflammatory responses (Phillips et al., 2004), which might render them hyper-responsive to differences in microbiomes associated with housing conditions or differences in glucocorticoid secretion associated with psychological stress. Increased cytokine secretion elicited by particular microbiomes and severe stress have also been shown to increase the phospho-tau load (Green et al., 2006; Sotiropoulos et al., 2011; Silva et al., 2019). Such changes in neuroinflammation and tau phosphorylation could easily render the P301S mice with TIA1 reduction extremely sensitive to housing conditions and elicit variability within cohorts and among experiments.

## Conclusion and Future Directions

This study provides multiple lines of convergent evidence that TIA1 reduction -both haploinsufficiency and knockout- strongly exacerbates typically deleterious aspects of neuroinflammation in the P301S mouse, including reactive microgliosis, antigen presentation, phagocytosis, pro-inflammatory cytokine production, and oxidative stress. These results are consistent with those observed in peripheral immunity, suggesting that TIA1 similarly acts as a translational repressor of inflammation in the central nervous system during stress. These findings are important given the key role TIA1 plays in the pathogenesis of tauopathy, and in ALS/FTD (Apicco et al., 2017; Hirsch-Reinshagen et al., 2017; Maziuk et al., 2018; Jiang et al., 2019). While reduction of TIA1 may lead to harmful cytotoxic inflammation and oxidative stress, it also restores microglial phagocytic function, which may prove to be beneficial in the context of tauopathy. To further characterize the relationship between TIA1 reduction, inflammation, and tauopathy, we have begun generating a conditional knock-out mouse model, in which TIA1 is specifically removed in either microglia or neurons in P301S mice. This model will help to interrogate the mechanisms underlying beneficial as well as detrimental effects of TIA1 reduction in tauopathy. Use of *in vitro* neuron and microglia co-culture models in future studies will enable direct measurement of the effects of TIA1 expression on the phagocytosis of phospho-tau. In providing novel evidence that TIA1 plays a key part in regulating the innate immune response of the CNS during stress, this study provides new avenues for interrogation of the pathogenesis of diseases in which neuroinflammatory processes play an important role.

## Data Availability Statement

The datasets generated for this study are available on request to the corresponding author.

## Ethics Statement

The animal study was reviewed and approved by the Institutional Animal Care and Use Committee at Boston University School of Medicine.

## Author Contributions

CL, BW, and JL: conceptualization. CL, NN, EH, JS, AC, JZ, and MM: performance of experiments. CL, NN, JZ, and MM: data analysis. BW and JL: funding acquisition. BW and JL: project administration. JL: supervision. MM: expertise. CL and JL: writing – original draft. CL, NN, EH, JS, AC, MM, BW, and JL: writing – review and editing.

## Conflict of Interest

BW is co-founder and Chief Scientific Officer for Aquinnah Pharmaceuticals Inc. The remaining authors declare that the research was conducted in the absence of any commercial or financial relationships that could be construed as a potential conflict of interest. The reviewer IS declared a past co-authorship with one of the authors, BW to the handling Editor.
