# Correction: Reduction of the RNA binding protein TIA1 exacerbates neuroinflammation in tauopathy

**DOI:** 10.3389/fnins.2025.1641304

**Published:** 2025-07-01

**Authors:** Chelsey Jenna LeBlang, Maria Medalla, Nicholas William Nicoletti, Emma Catherine Hays, James Zhao, Jenifer Shattuck, Anna Lourdes Cruz, Benjamin Wolozin, Jennifer Irene Luebke

**Affiliations:** ^1^Laboratory of Cellular Neuroscience, Department of Anatomy & Neurobiology, Boston University School of Medicine, Boston, MA, United States; ^2^Laboratory of Neurodegeneration, Department of Pharmacology & Experimental Therapeutics, Boston University School of Medicine, Boston, MA, United States; ^3^Department of Neurology, Boston University School of Medicine, Boston, MA, United States; ^4^Department of Neuroscience, Boston University, Boston, MA, United States

**Keywords:** tauopathy, neuroinflammation, microglia, hippocampus, RNA binding protein, neurodegeneration

There was a mistake in Figure 4D, Row 2, Column 4 as published. P301S +/+ 9M image was duplicated in error under P301S TIA1 -/- 9M. The corrected Figure 4 appears below.

**Figure 4:**
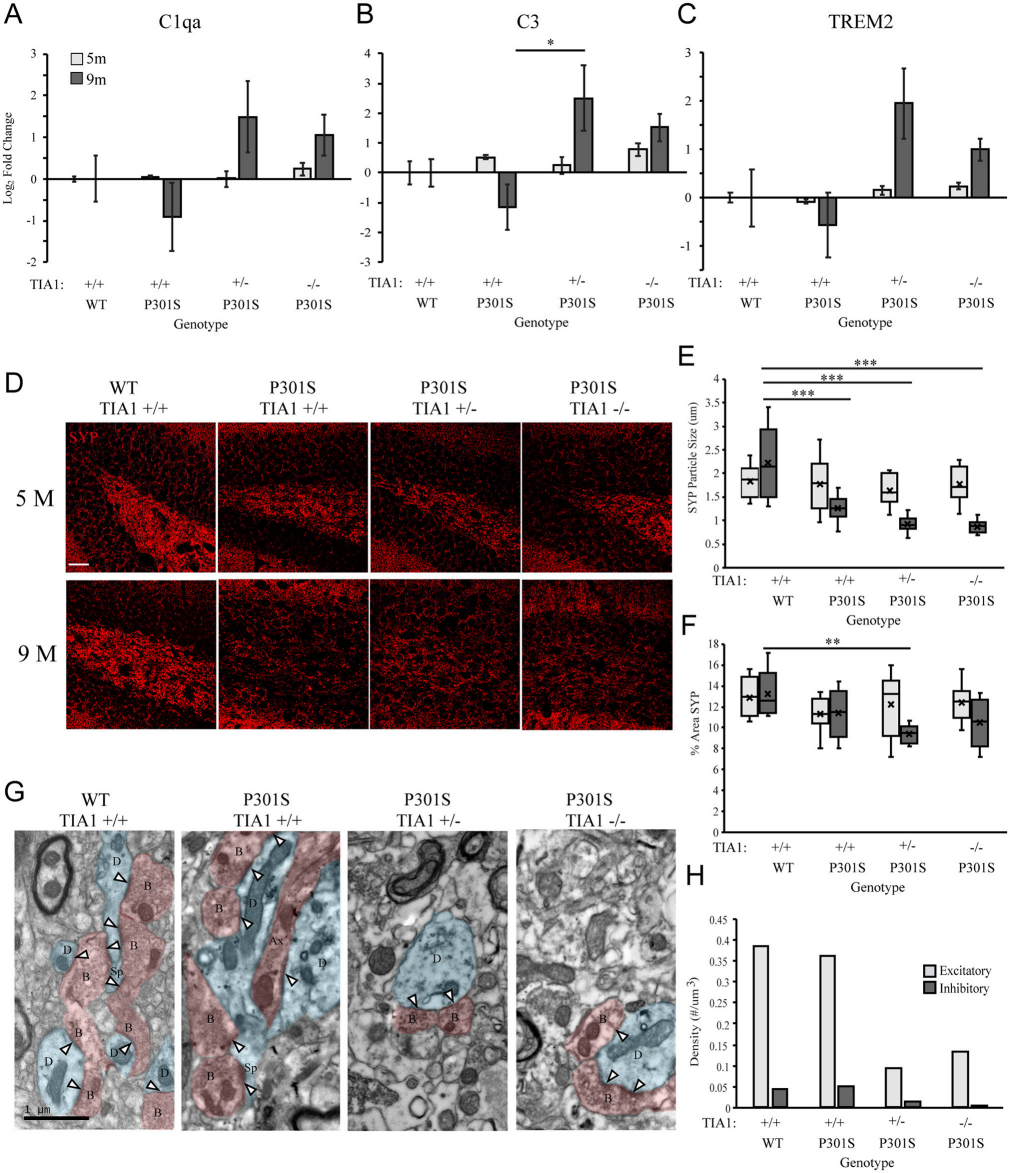
Reduction of TIA1 increases expression of mRNAs associated with microglial phagocytosis and increases synapse loss in advanced tauopathy. **(A)** log2 fold change of C1qa expression in WT TIA1+/+ vs. P301S TIA1+/+, P301S TIA1+/−, and P301S TIA1−/− at 5 months (light gray) and 9 months (dark gray). n = 3 subjects/genotype. **(B)** log2 fold change of C3 expression in WT TIA1+/+ vs. P301S TIA1+/+, P301S TIA1+/−, and P301S TIA1−/− at 5 months (light gray) and 9 months (dark gray). n = 3 subjects/genotype. **(C)** log2 fold change of TREM2 expression in WT TIA1+/+ vs. P301S TIA1+/+, P301S TIA1+/−, and P301S TIA1−/− at 5 months (light gray) and 9 months (dark gray). n = 3 subjects/genotype. **(D)** High resolution (40×) confocal maximum projection images depicting Synaptophysin (SYP, red) in the apex of the dentate gyrus including the dorsal and ventral dentate granule cell layers and hilus in WT TIA1+/+ and P301S subjects that are TIA1+/+, TIA1+/−, and TIA1−/− at 5 months (top) and 9 months (bottom). Scale = 25 μm. **(E)** Average size of SYP particles in **(D)**, 5 months (light gray) and 9 months (dark gray). n = 9–12 fields, from 3 to 4 animals/genotype. **(F)** Percent Area occupied by SYP particles in **(D)**, 5 months (light gray) and 9 months (dark gray). n = 9–12 fields, from 3 to 4 animals/genotype. **(G)** Electron photomicrographs at 20k × depicting synapses (Presynaptic structures in red, B, bouton; Ax, axon; Postsynaptic structures in blue, D, dendrite; Sp, spine; Synaptic contacts = white arrowheads) in the dentate gyrus hilus neuropil in WT TIA1+/+ and P301S subjects that are TIA1+/+, TIA1+/−, and TIA1−/− animals. Scale = 1 μm. **(H)** Average density of excitatory (light gray) and inhibitory (dark gray) synapses in the dentate gyrus hilus neuropil in WT TIA1+/+ and P301S TIA1+/+, TIA1+/−, and TIA1−/− animals. (n = 3–4 fields, from 1 animal/genotype, no statistical analyses performed, qualitative data). All statistical comparisons calculated with One-Way ANOVA with Bonferroni post hoc test, *p ≤ 0.05, **p ≤ 0.01, ***p ≤ 0.001.

The original article has been updated.

